# Designing single trigger/dual-response release and degradation into amine-functional hyperbranched-polydendron nanoprecipitates†

**DOI:** 10.1039/d0na00696c

**Published:** 2020-10-28

**Authors:** Hannah E. Rogers, Pierre Chambon, Sean Flynn, Faye Y. Hern, Andrew Owen, Steve P. Rannard

**Affiliations:** Department of Chemistry, University of Liverpool Crown Street L69 7ZD UK srannard@liverpool.ac.uk; Materials Innovation Factory, University of Liverpool Crown Street L69 7ZD UK; Department of Molecular and Clinical Pharmacology, University of Liverpool Block H, 70 Pembroke Place Liverpool L69 3GF UK

## Abstract

The synthesis of complex polymer architectures using relatively facile experimental protocols provides access to materials with the opportunity to control functionality and physical behaviour. The scope of hyperbranched-polydendron chemistries has been expanded here to include primary chains comprising amine-functional ‘homopolymer’, ‘statistical copolymer’ and amphiphilic ‘block copolymer’ analogues using 2-(diethyl amino)ethyl methacrylate, 2-hydroxy propyl methacrylate and *t*-butyl methacrylate. The different primary chain chemistry and architectures leads to a marked variation in nanoprecipitation behaviour and the response of the resulting amine-functional nanoparticles to varying pH. When acid-sensitive and acid-stable branchers, 1,4-butanediol di(methacryoyloxy)-ethyl ether and ethylene glycol dimethacrylate respectively, are utilised, nanoparticles with encapsulation properties are formed and may be triggered to either release-and-disassemble or release-disassemble-degrade to form a solution of lower molecular weight constituent primary chains.

## Abstract 2

The synthesis of complex polymer architectures using relatively facile experimental protocols provides access to materials with the opportunity to control functionality and physical behaviour.

## Graphical abstract

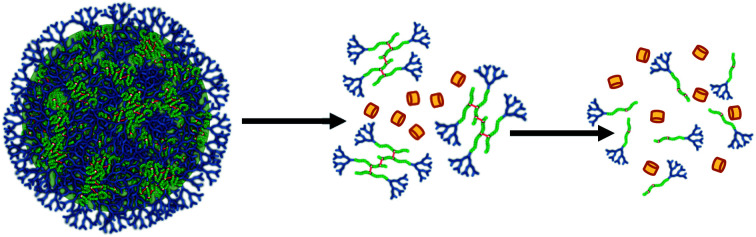

## Introduction

Stimuli-responsive materials have been of considerable interest for many applications and the formation of defined polymeric structures has been at the core of many research activities to enable fine control of material behaviour.^1^ Advances have led to studies of materials that are programmable,^2^ exhibit multiple responses in the solid state,^3^ respond to stimuli in solution,^4^ and self-assemble when triggered by a range of environmental factors.^5^ A number of recurring themes are present within the wealth of reports of stimuli-responsive polymeric materials; these include the use of branched^6^ and segmented polymer^7^ architectures, the manipulation of aqueous solution pH^8^ to drive amine-functional polymer behaviour, and the ability to encapsulate and release a payload material from a nanoscale self-assembled object.^9^

Amine methacrylate monomers^10^ have been widely used to create pH-responsive polymer structures, led in part by the Armes group; their reports of shell-crosslinked micelles created from A–B diblock^11^ and A–B–C triblock^12,13^ copolymers exploited the manipulation of hydrophilicity within the different block segments using pH, temperature and electrolyte concentrations. The study of amine-methacrylate derived pH-responsive branched vinyl copolymers, synthesised by the so-called “Strathclyde” route,^14^ showed the reversible formation of sterically-stabilised nanoparticles during the manipulation of solution pH. The relatively high molecular weight copolymers were lightly branched with low molar concentrations of ethylene glycol dimethacrylate (EGDMA) under conventional free radical conditions and also contained oligo(ethylene glycol) methacrylate to prevent macroscale phase separation and aggregation.^15^

Encapsulation, release and degradation studies of assembled materials offers the potential to create delivery vehicles that may enhance the formulation capability and efficacy of small molecules in a range of applications from pharmaceutical drug delivery through to agrochemicals, antimicrobials and cosmetics.^16^ In each of these cases, the degradability of the building block structures to smaller subunits that may be benign, either in an *in vivo* context or in the wider environment, is very important and has been the aim of materials such as self-immolative polymers^17,18^ and triggers including reduction,^19^ hydrolysis,^20^ temperature,^21^ chemical,^22^ and light enhanced^23^ mechanisms.

In recent years, we have introduced a new copolymer architecture that combines the concepts of branched vinyl copolymerisation and linear-dendritic hybrids^24^ (LDH; linear polymers with ideal dendron chain ends), using low concentrations of divinyl monomers^25^ within the controlled radical polymerisation of the LDH structures. The resulting copolymers, named hyperbranched-polydendrons (*hyp*-PDs), have a complex architecture of large numbers of conjoined chains with ideally-branched, highly functional dendron chain ends.^26–28^ We have shown that despite the broad dispersity (*Ð*) of *hyp*-PDs, self-assembly under flash nanoprecipitation conditions rapidly generates highly uniform nanoparticles that bear dendron functionality at the surface; the process is mediated by the very high molecular weight component of the molecular weight distribution.^29^ In all examples of *hyp*-PDs to date, hydrophobic monomers have comprised the primary polymer chains of the branched polymer structure, although dendrons of varying functionality and scaffold chemistry have been utilised. Here we present a fundamental study of the introduction of 2-(diethyl amino)ethyl methacrylate (DEAEMA) into the formation of *hyp*-PDs with the direct aim of achieving a single trigger/dual-response release and degradation of pH-responsive nanoprecipitates. A number of comparative structures have been synthesised that allow the testing of our initial hypotheses and an expansion in the understanding of the architectural variation available from the *hyp*-PD polymer platform.

## Results and discussion

### Design and preparation of pH-responsive 2-(diethyl amino)ethyl methacrylate containing hyperbranched-polydendron structures

**Fig. 1:**
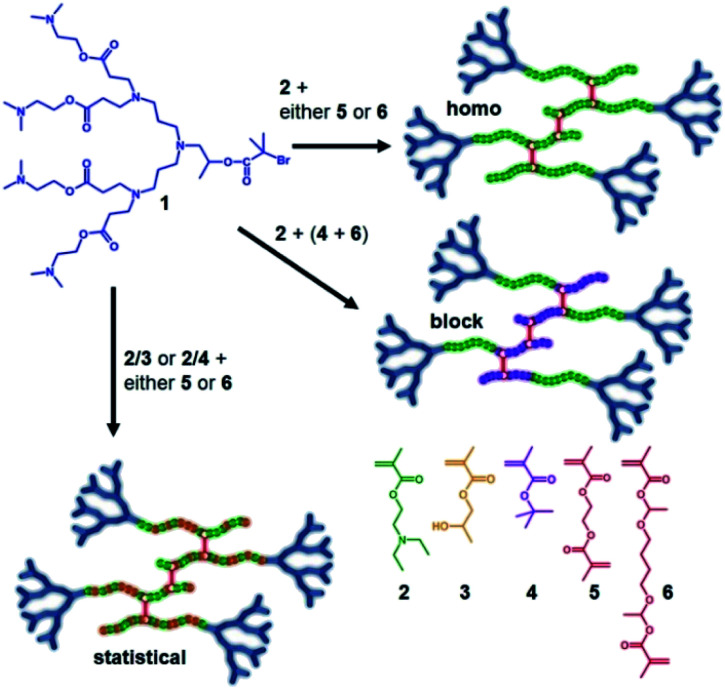
Schematic illustration of the target responsive hyperbranched-polydendron materials for this study (four conjoined primary chains shown in the simplified structures).

Our previous reports of *hyp*-PDs have largely relied upon hydrophobic monomers such as 2-hydroxy propyl methacrylate (HPMA),^30^*n*-butyl methacrylate^31^ and *t*-butyl methacrylate (*t*BuMA)^27^ to encourage nanoprecipitation of the resulting materials. Copper catalysed atom transfer radical polymerisation (ATRP) has been successfully employed to generate *hyp*-PDs after the formation and use of tertiary-bromide functional dendron initiators; pH-responsive behaviour has only previously been studied using hydrophobic monomers and tertiary-amine functional dendron initiators such as the second generation (G_2_) dendron initiator, 1, employed here (Fig. 1, see ESI Fig. S1†).^32^

The materials targeted for this study utilise 1 to initiate the ATRP of either (a) DEAEMA, 2, (b) monomer mixtures of DEAEMA/HPMA, 3, or DEAEMA/*t*BuMA, 4, or (c) blocks of DEAMEA and *t*BuMA. Two divinyl branching monomers were selected, namely EGDMA, 5, which would not be expected to demonstrate any pH response, and 1,4-butanediol di(methacryoyloxy)-ethyl ether (BDME, 6), expected to be cleaved under acidic pH conditions. BDME was synthesised according to a minor modification of the previously reported synthesis of ethylene glycol di(1-methacryloyloxy)ethyl ether (see ESI Fig. S2–S4†).^33^ The copolymerisation of DEAEMA with either EGDMA or BDME yields a *hyp*-PD with primary chains that are dominated by the homopolymer of DEAEMA, denoted here as a “homo *hyp*-PD” (Fig. 1). Similarly, DEAEMA/HPMA or DEAEMA/*t*BuMA mixtures create statistical copolymer primary chains, denoted as a “statistical *hyp*-PD”, and the sequential propagation of DEAEMA followed by a mixture of *t*BuMA with either EGDMA or BDME will create predominantly A–B block copolymer primary chains, denoted here as a “block *hyp*-PD” (Fig. 1).

### Hyperbranched-polydendron synthesis comprising 2-(diethyl amino)ethyl methacrylate and ethylene glycol dimethacrylate

**Fig. 2:**
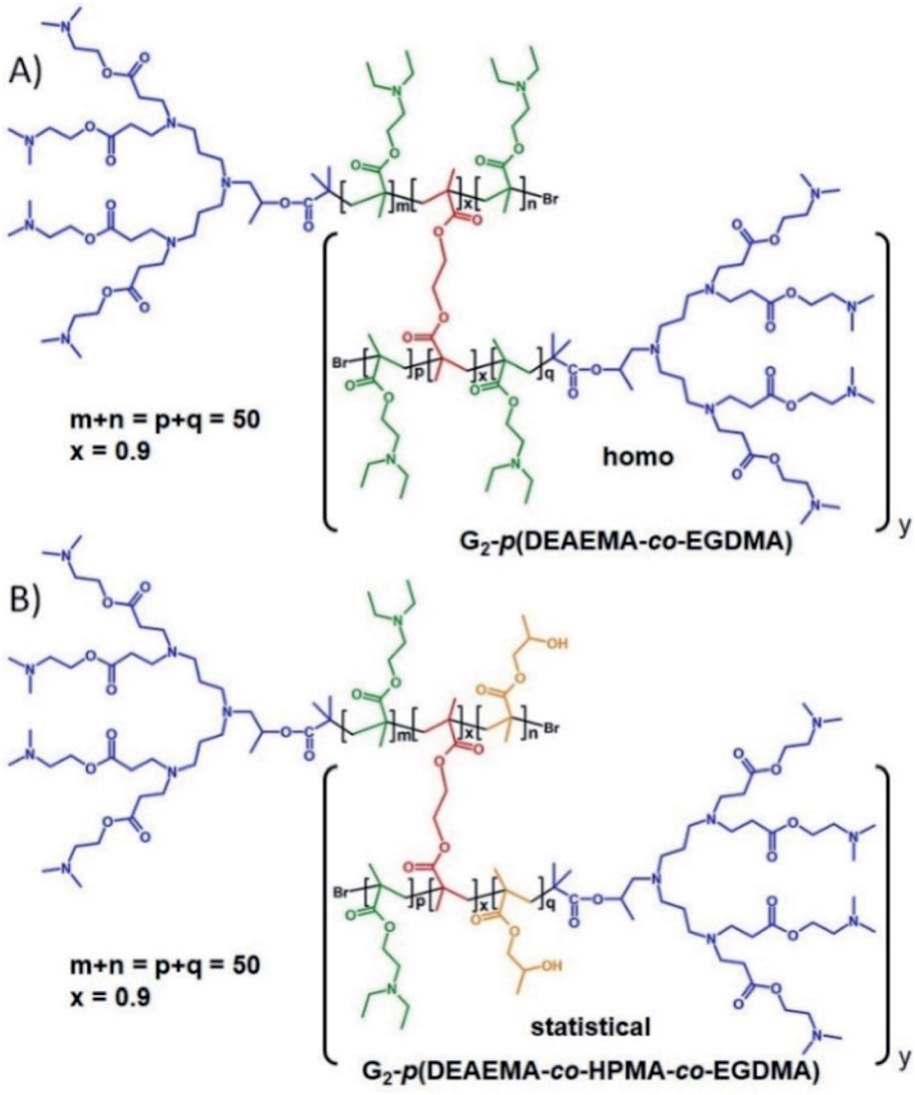
Homo and statistical hyperbranched-polydendron chemical structure examples using the G_2_ amine functional dendron initiator, 1. (A) Homo *hyp*-PD comprising poly(2-(diethyl amino)ethyl methacrylate), DEAEMA, within the primary chains using ethylene glycol dimethacrylate, EGDMA, as the branching agent; (B) statistical *hyp*-PD comprising DEAEMA, EGDMA and 2-hydroxy propyl methacrylate within the primary chains.

The ATRP synthesis of the homo *hyp*-PD structure using 1, DEAEMA and EGDMA (Fig. 2A) was conducted at 40 °C in a isopropanol/water mixture (IPA/H_2_O; 92.5 : 7.5%, 39 wt% monomer) and progressed as reported for other monovinyl-methacrylate monomers using a Cu(i)Cl/bpy catalyst system (1 : 2).

Each primary chain targeted a number average degree of polymerisation (DP_*n*_) of 50 monomer units and at this relatively low solids content, a degree of intramolecular cyclisation would be expected as previously reported;^34^ the reaction concentration was somewhat dictated by the balance of solubilities of the different reaction components. Polymerisations in the absence of EGDMA, to form the LDH structures (DP_*n*_ = 50 monomer units), were also conducted to establish the appropriate conditions and enable estimations of the structure of the final complex architectures (see ESI Fig. S2–S4†)

The introduction of HPMA or *t*BuMA into the DEAEMA monomer feedstock led to no noticeable complications within the formation of the statistical LDH copolymer, or for the statistical *hyp*-PD when EGDMA was also incorporated (see ESI Fig. S5–S11†). The ratio of DEAEMA/HPMA or DEAEMA/*t*BuMA was varied from 33/17 to 25/25 and 17/33, each targeting a DP_*n*_ = 50 monomer units, in both the statistical LDH and statistical *hyp*-PD structures. The triple detection size exclusion chromatography (TD-SEC) analysis of all homo- and statistical LDH and *hyp*-PDs is shown in Table 1. All *hyp*-PDs utilised a [1] : [EGDMA] ratio of 1.0 : 0.9 to avoid gelation and maintain an average of less than one brancher per chain.

**Table 1:** Size exclusion chromatography analysis of linear-dendritic hybrids, homo and statistical hyperbranched polydendrons

Polymer	*M*_n_ theory (g mol^−1^)	TD-SEC^a^		*Ð*
		*M*_n_ (g mol^−1^)	*M*_w_ (gmol^−1^)	
**LDH**				
G_2_-*p*(DEAEMA_50_)	10 090	23 650	34 400	1.45
G_2_-*p*(DEAEMA_33_-*co*-HPMA_17_)	9400	11 750	14 600	1.24
G_2_-*p*(DEAEMA_25_-*co*-HPMA_25_)	9070	12 600	16 550	1.31
G_2_-*p*(DEAEMA_17_-*co*-HPMA_33_)	8740	12 200	16 000	1.31
G_2_-*p*(DEAEMA_33_-*co*- *t*BuMA_17_)	9360	9000	10 350	1.15
G_2_-*p*(DEAEMA_25_-*co*- *t*BuMA_25_)	9020	9800	11 350	1.16
G_2_-*p*(DEAEMA_17_-*co*- *t*BuMA_33_)	8670	9850	11 050	1.12

***hyp*-PDs**				
G_2_-*p*(DEAEMA_50_-*co*-EGDMA_0.9_)	—	125 700	341 800	2.72
G_2_-*p*(DEAEMA_33_-*co*-HPMA_17_-*co*-EGDMA_0.9_)	—	247 500	398 300	1.61
G_2_-*p*(DEAEMA_25_-*co*-HPMA_25_-*co*-EGDMA_0.9_)	—	268 900	1 971 000	7.33
G_2_-*p*(DEAEMA_17_-*co*-HPMA_33_-*co*-EGDMA_0.9_)	—	682 000	4 510 000	6.61
G_2_-*p*(DEAEMA_33_-*co-t*BuMA_17_ -*co*-EGDMA_0.9_)	—	209 200	392 400	1.88
G_2_-*p*(DEAEMA_25_-*co-t*BuMA_25_-*co*-EGDMA_0.9_)	—	67 200	140 900	2.10
G_2_-*p*(DEAEMA_17_-*co-t*BuMA_33_-*co*-EGDMA_0.9_)	—	149 200	531 100	3.56

a Triple detection size exclusion chromatography using THF/2% TEA eluent; all polymerisation attained >99% monomer conversion as determined by ^1^H NMR.

**Fig. 3:**
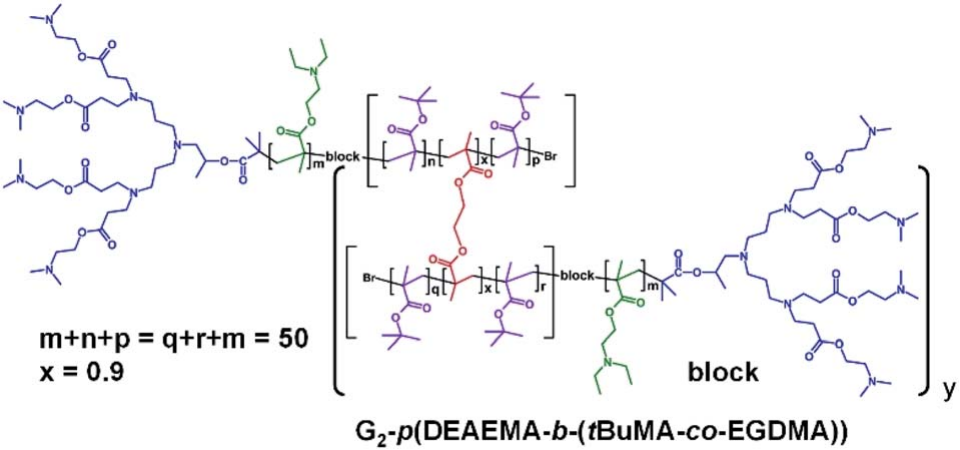
Chemical structure example of block hyperbranched-polydendron using the G_2_ amine functional dendron initiator, 1, 2-(diethyl amino)ethyl methacrylate and *t*-butyl methacrylate within the primary chains.

The synthesis of block *hyp*-PDs was undertaken after a series of block LDH structures containing various molar ratios of DEAEMA and *t*BuMA had been successfully demonstrated (Table 2, see ESI Fig. S12 and S13†); this represents the first report for the formation of such structures (Fig. 3).

**Table 2:** Size exclusion chromatography analysis of linear-dendritic hybrids and block hyperbranched polydendrons

Polymer	*M*_n_ theory (g mol^−1^)	TD-SEC^a^		*Ð*
		*M*_n_ (g mol^−1^)	*M*_w_ (gmol^−1^)	
**LDH**				
G_2_-*p*(DEAEMA_33_-*b-t*BuMA_17_)	9360	35 400	40 850	1.15
G_2_-*p*(DEAEMA_25_-*b-t*BuMA_25_)	9020	33 000	46 450	1.40
G_2_-*p*(DEAEMA_17_-*b-t*BuMA_33_)	8670	40 350	45 100	1.12

***hyp*-PDs**				
G_2_-*p*(DEAEMA_33_-*b*-(*t*BuMA_17_-*co*-EGDMA_0.9_))	—	86 900	192 100	2.21
G_2_-*p*(DEAEMA_25_-*b*-(*t*BuMA_25_-*co*-EGDMA_0.9_))	—	265 400	528 900	1.99
G_2_-*p*(DEAEMA_17_-*b*-(*t*BuMA_33_-*co*-EGDMA_0.9_))	—	92 300	251 800	2.73

**Comparison**				
G_2_-*p*(*t*BuMA_50_)	7940	6900^b^	7650	1.11
G_2_-*p*(*t*BuMA_50_-*co*-EGDMA_0.95_)	—	88 200	273 300	3.10

a Triple detection size exclusion chromatography using THF/2% TEA eluent; all polymerisation attained >98% monomer conversion as determined by ^1^H NMR.

b Polymerisation attained 94% conversion (*M*_n_ theory @ 94% = 7460 g mol^−1^)

Again, ATRP polymerisation in IPA/H_2_O was conducted at 40 °C initially synthesising the DEAEMA block segment before addition of *t*BuMA or the *t*BuMA/EGDMA mixture at approximately 80% conversion to form a block LDH or block *hyp*-PD respectively; in this way, branching was restricted to the second hydrophobic block segment with limited mixing of the block chemistries.^35^ For consistency, block copolymers with a total (A + B) block length of DP_*n*_ = 50 monomer units were targeted and the DEAEMA/*t*BuMA ratio was varied from 33/17 to 25/25 and 17/33 to match the ratios within the statistical *hyp*-PD structures.

It is clear from the SEC data (Tables 1 and 2) that the initiator efficiency (IE) of 1 is relatively low when used with this amine-containing methacrylate monomer, as we also reported for HPMA homopolymerisation previously.^32^ When DEAEMA is polymerised solely, this appears to be approximately 50% (as indicated by a comparison of targeted and experimentally determined number average molecular weight (*M*_n_)), but the inclusion of comonomers within the synthesis of statistical copolymerisation appears to lead to better targeting of DP_*n*_ (IE > 70%) and *Ð* values are consistently below 1.32 (<1.17 when *t*BuMA present). Low initiator efficiency will lead to experimental [1] : [EGDMA] ratios > 1 per chain, however this has been compensated by intramolecular cyclisation that would be expected at these concentrations, as discussed above.

The purified homo and statistical *hyp*-PD samples (Table 1) have a considerable weight average number of conjoined primary chains, ranging from 14–370 chains, calculated as the ratio of weight average molecular weight (*M*_w_) of the branched polymer and the *M*_n_ of the corresponding LDH synthesised without brancher, *i.e. M*_w_(*hyp*-PD)/*M*_n_(LDH). This increases with decreasing DEAEMA, as does *M*_w_ generally, and may indicate a negative impact of DEAEMA on the ATRP reaction under these conditions. Given that each primary chain-end bears 7 tertiary amine functional groups these materials comprise, on a weight average basis, between 98 and >2500 dendron-derived tertiary amines at the periphery of the complex architecture.

The block *hyp*-PDs (Table 2) rely upon the homopolymerisation of DEAEMA within the first linear block segment to form an external amine-functional block segment, and are subject to the low initiator efficiency of the homopolymerisation as described above; however, relatively low dispersity LDH block copolymers were synthesised in the absence of EGDMA and high molecular weight block *hyp*-PDs were synthesised, as expected, when EGDMA was included in the formation of the second block. As described above, the weight average number of chains for the block *hyp*-PD samples varied from 5–16 conjoined primary chains, and the structures therefore bear between 35 and 112 peripheral dendron-derived tertiary amine groups. In order to investigate the impact of DEAEMA on the initiator efficiency of 1, the LDH of *t*BuMA was synthesised (Table 2; see ESI Fig. S14 and S15†) and close targeting of *M*_n_ with low *Ð* was observed; the corresponding homo *hyp*-PD G_2_-*p*(*t*BuMA_50_-*co*-EGDMA_0.95_) was synthesised using a higher EGDMA content, with a weight average number of 40 conjoined primary chains (see ESI Fig. S16†).

### Synthesis and acid degradation of hyperbranched-polydendrons comprising 2-(diethyl amino)ethyl methacrylate and BDME, 6

The synthesis of BDME-containing *hyp*-PDs was conducted using a primary chain DP_*n*_ = 50 monomer units, for consistency; however, the incorporation of BDME was not as efficient as EGDMA and a 1 : BDME molar ratio of 1 : 2 was required to generate high molecular weight materials. This may again be due to significant intramolecular cyclisation and a reduction in the intermolecular branching by BDME as described above. At a 1 : 2 molar ratio, three polymer structures were targeted as comparisons with EGDMA-containing *hyp*-PDs described above with all syntheses reaching >99% monomer conversion (Table 3, see Fig. S17 and S18†); the selection of these structures is explained later.

**Table 3:** Size exclusion chromatography analysis of homo, statistical and block hyperbranched polydendrons synthesised using 1,4-butanediol di(methacryoyloxy)-ethyl ether, 6

Polymer	SEC^a^		*Ð*
	*M*_n_ (g mol^−1^)	*M*_w_ (gmol^−1^)	
***hyp*-PDs**			
G_2_-*p*(DEAEMA_50_-*co*-BDME_2.0_)	157 300	321 100	2.04
G_2_-*p*(DEAEMA_33_-*co*-HPMA_17_-*co*-BDME_2.0_)	305 300	733 400	2.40
G_2_-*p*(DEAEMA_17_-*b*-(*t*BuMA_33_-*co*-BDME_2.0_))	444 100	594 800	1.34

a Triple detection size exclusion chromatography using THF/2% TEA eluent; all polymerisation attained >99% monomer conversion as determined by ^1^H NMR.

The introduction of an acid-sensitive brancher allows the triggering of branched copolymer degradation under acid conditions. The susceptibility of the BDME linking chemistry to HCl was confirmed by dissolving the three *hyp*-PD architectures containing BDME in acetone prior to dropwise addition of 1 M HCl at ambient temperature.

**Fig. 4:**
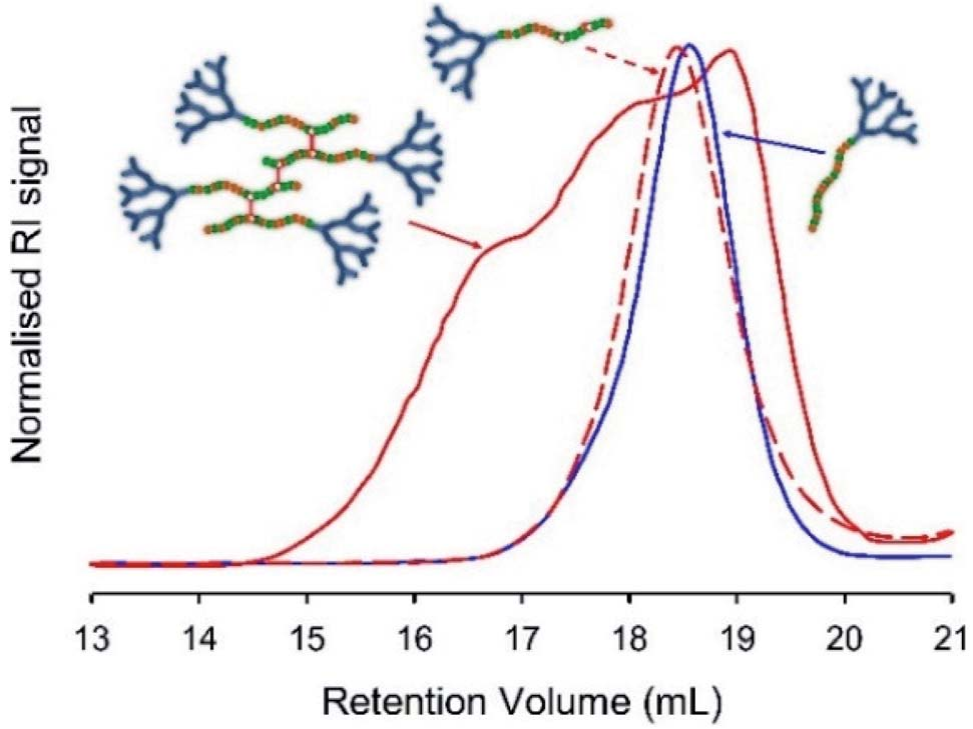
Illustrative example of TD-SEC analysis (refractive index) of pH-triggered degradation of BDME containing hyperbranched-polydendrons. Example shows statistical *hyp*-PD G_2_-*p*(DEAEMA_33_-*co*-HPMA_17_-*co*-BDME_2.0_) before addition of HCl (red solid line), the sample after acid addition and purification (red dotted line) and the corresponding G2-*p*(DEAEMA_33_-*co*-HPMA_17_) statistical linear-dendritic hybrid (blue solid line) for comparison.

Freeze-drying was used to remove all solvents, and the resulting polymer samples were dissolved in THF/TEA (2% v/v) and compared with the corresponding LDH samples of near identical composition (Table 1), and the starting *hyp*-PD structure, using SEC. In principle, the cleavage of the BDME link would lead to the formation of a polymer distribution comprising LDH structures of very similar structure and molecular weight to the LDH polymers formed in the absence of brancher (Fig. 4, see ESI Fig. S19 andS20†); this was indeed observed for each of the homo, statistical and block structures and further supports the conjoined primary chain nature of the *hyp*-PD structure and is analogous to studies conducted on non-dendron bearing branched polymers.^36^

### Nanoprecipitation of amine-functional EGDMA-containing hyperbranched-polydendrons of varying primary chain structure

As mentioned above, nanoprecipitation of *hyp*-PDs has been shown to benefit from a low concentration of structures containing large numbers of conjoined primary chains. In our earlier reports, a simple rapid nanoprecipitation approach was remarkably successful in forming stable nanoparticles with narrow distributions; we have utilised a similar process here, however, the presence of amine functionality within the dendron chain-end and primary chains does provide the opportunity to study nanoprecipitation within aqueous anti-solvent environments of different pH. To standardise the rapid nanoprecipitation process for the materials containing EGDMA, each polymer was dissolved in acetone (good solvent) at a concentration of 5 mg mL^−1^ and 2 mL of the solution was rapidly added to 10 mL of stirred water (anti-solvent); after overnight solvent removal *via* evaporation, the resulting aqueous nanoparticle dispersion was at a concentration of 1 mg mL^−1^, as reported previously. Samples were unfiltered and all studies were undertaken on unpurified aqueous dispersions.

The nanoprecipitation of each *hyp*-PD was studied by following this approach using deionised water as the bad solvent phase at a pH = 7.8 (Table 4). The G_2_-*p*(DEAEMA_50_-co-EGDMA_0.9_) *hyp*-PD generated a narrow dispersity sample (PDI = 0.065) with a z-average diameter (*D*_z_) of 60 nm and a zeta potential (*ζ*) of +22 mV as determined by dynamic light scattering (DLS) and electrophoretic mobility studies. The positive zeta potential was expected due to protonation of the amine functional groups within this *hyp*-PD. The statistical *hyp*-PDs based on G_2_-*p*(DEAEMA_*x*_-*co*-HPMA_*y*_-*co*-EGDMA_0.9_) were also nanoprecipitated under these conditions leading to particle dispersions of larger *D*_z_, ranging between 115–145 nm (see ESI Fig. S21†), but maintaining relatively narrow distributions (PDI = 0.099–0.166) with the most monodisperse dispersion being formed from polymers with the highest HPMA content; this also correlates to the highest *M*_n_ and *M*_w_ values and again indicates the importance of having large numbers of conjoined primary chains as we have reported previously.^29^ Within these dispersions a low zeta potential was observed, correlating to the reduction in DEAEMA content as may be expected (*ζ* = 13–16 mV) with the lowest value being obtained for the highest HPMA content.

**Table 4:** Dynamic light scattering analysis of nanoprecipitates formed from various homo, statistical and block hyperbranched-polydendrons under different pH conditions and after treatment with aqueous HCl

Polymer	Dynamic light scattering data									
	Initial pH 4.0 (final pH = 5.9–7.4)^a^			Initial pH 7.8 (final pH = 6.9–7.9)^b^				Acid addition (final pH = 2.6–3.1)^c^		
	*D*_z_	PDI	*D*_n_	*D*_z_	PDI	*D*_n_		*D*_z_	PDI	*D*_n_
G_2_-*p*(DEAEMA_50_-*co*-EGDMA_0.9_)	is	is	is	60	0.065	45	→	is	is	is
G_2_-*p*(DEAEMA_33_-*co*-HPMA_17_-*co*-EGDMA_0.9_)	—	—	—	145	0.113	105	→	is	is	is
G_2_-*p*(DEAEMA_25_-*co*-HPMA_25_-*co*-EGDMA_0.9_)	—	—	—	185	0.166	140		is	is	is
G_2_-*p*(DEAEMA_17_-*co*-HPMA_33_-*co*-EGDMA_0.9_)	—	—	—	115	0.099	80		is	is	is
G_2_-*p*(DEAEMA_33_-*co-t*BuMA_17_-*co*-EGDMA_0.9_)	50	0.222	20	u	u	u	→	is	is	is
G_2_-*p*(DEAEMA_25_-*co-t*BuMA_25_-*co*-EGDMA_0.9_)	135	0.284	35	u	u	u		is	is	is
G_2_-*p*(DEAEMA_17_-*co-t*BuMA_33_-*co*-EGDMA_0.9_)	130	0.222	60	u	u	u		141	0.293	51
G_2_-*p*(DEAEMA_33_-*b*-(*t*BuMA_17_-*co*-EGDMA_0.9_))	is	is	is	u	u	u	→	is	is	is
G_2_-*p*(DEAEMA_25_-*b*-(*t*BuMA_25_-*co*-EGDMA_0.9_))	is	is	is	u	u	u		is	is	is
G_2_-*p*(DEAEMA_17_-*b*-(*t*BuMA_33_-*co*-EGDMA_0.9_))	85	0.197	45	u	u	u		155	0.354	77
G_2_-*p*(*t*BuMA_50_-*co*-EGDMA_0.95_)	235	0.295	80	u	u	u	→	—	—	—
G_2_-*p*(DEAEMA_50_-*co*-BDME_2.0_)	—	—	—	60	0.241	30	→	is	is	is
G_2_-*p*(DEAEMA_33_-*co*-HPMA_17_-*co*-BDME_2.0_)	—	—	—	410	0.204	310		is	is	is
G_2_-*p*(DEAEMA_17_-*b*-(*t*BuMA_33_-*co*-BDME_2.0_))	—	—	—	110	0.086	75		135	0.073	105

a Data from nanoprecipitating into aqueous HCl at pH = 4.0.

b Data from nanoprecipitating into deionised water at pH = 7.8.

c Data obtained after treating nanoprecipitates with aqueous HCl and forming a final pH = 2.6–3.1. is = insufficient scattering (suggests solvation); — = experiment not conducted; u = unsuccessful experiment.

The statistical *hyp*-PDs containing *t*BuMA did not nanoprecipitate into water at pH = 7.8 and neither did the block *hyp*-PDs (Table 4). It is interesting that our previous nanoprecipitation reports of HPMA-derived materials (without amine functionality) led to stable nanoparticles with highly negative *ζ* values when nanoprecipitated in this way, and here we see very different behaviour when *t*BuMA is employed. For comparison, we synthesised the homo *hyp*-PD G_2_-*p*(*t*BuMA_50_-*co*-EGDMA_0.95_) (Table 2), and this also failed to nanoprecipitate under these conditions. In order to study this in greater detail, the range of statistical, block and homopolymer *hyp*-PDs containing *t*BuMA were nanoprecipitated into water containing HCl at pH = 4; the hypothesis underpinning this approach suggested the protonation of the amine functionality during nanoprecipitation may encourage colloidal stability and the unbuffered water would potentially lead to nearly neutral conditions after polymer addition and removal of solvent. As expected, the neutralisation during nanoprecipitation led to final pH values ranging from 5.9–7.4, depending on the branched copolymer composition, but the success of this approach varied quite considerably across the range of materials used.

**Fig. 5:**
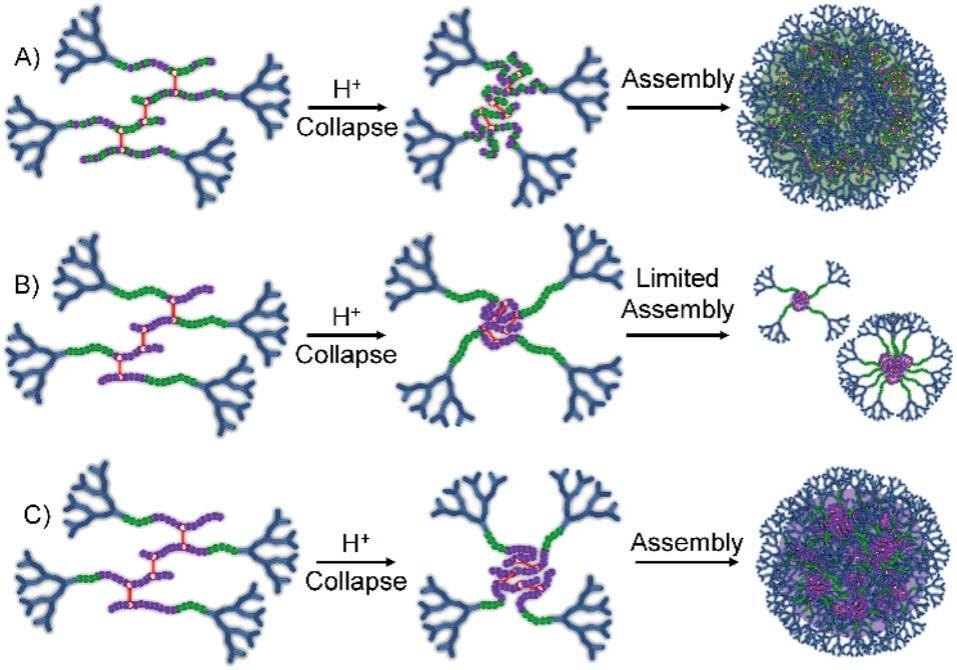
Schematic representation of the behaviour of statistical and block hyperbranched-polydendrons containing varying *t*-butyl methacrylate (*t*BuMA) monomer residue content during nanoprecipitation into acidic water. (A) Statistical *hyp*-PD with high *t*BuMA content; (B) block *hyp*-PD with short *t*BuMA block length; (C) block *hyp*-PD with long *t*BuMA block length.

G_2_-*p*(DEAEMA_50_-*co*-EGDMA_0.9_) was unable to nanoprecipitate under these conditions, leading to a clear and homogeneous solution; however, the statistical G_2_-*p*(DEAEMA_*x*_-*co-t*BuMA_*y*_-*co*-EGDMA_0.9_) polymer series generated small nanoparticles (*D*_z_ = 50 nm) at low *t*BuMA content and larger particles (*D*_z_ approximately 130 nm) at higher *t*BuMA ratios (Fig. 5A, see ESI Fig. S22†). Similar behaviour was seen for block *hyp*-PDs G_2_-*p*(DEAEMA_*x*_-*b*-(*t*BuMA_*y*_-*co*-EGDMA_0.9_)) but at the lowest block lengths of *t*BuMA (DP_*n*_ = 17 or 25 units), insufficient light scattering was seen, suggesting no precipitation and high levels of solvation. At the highest hydrophobic block length (DP_*n*_ = 33 monomer units), nanoprecipitation was observed (see ESI Fig. S23†) with nanoparticles of relatively small size formed (*D*_z_ = 85 nm).

This variation in behaviour between the chemically similar but architecturally different materials is notable and suggests a difference in hydrophobicity between the statistically mixed and segmented chemistries. Although we have not confirmed this rationale, it seems plausible that the block *hyp*-PDs assemble into stabilised unimolecular structures, or small aggregates (Fig. 5B), when the block length is short and the protonated dendron-terminated DEAEMA block dominates the behaviour during addition to the low pH aqueous environment; solubility behaviour within amphiphilic block structures is known to be composition and block length dependent.^37^ As the hydrophobic block length increases to 66% of the total primary chain, a switch to *t*BuMA block dominance is seen and a more conventional nanoprecipitation appears to occur (Fig. 5C); this observation determined the selection of the block *hyp*-PD structure G_2_-*p*(DEAEMA_17_-*b*-(*t*BuMA_33_-*co*-BDME_2.0_)) for studies using acid sensitive branchers.

Within the statistical copolymers, the mixing of DEAEMA and *t*BuMA leads to a clear impact on the solution behaviour of the protonated DEAEMA chain, even at 34 mole % *t*BuMA, and larger nanoparticles are formed as the *t*BuMA content increases, leading to an additional decrease in protonated amine functional groups and subsequent surface charge density of the protonated macromolecules; the *t*BuMA-containing nanoprecipitates from this statistical copolymer series exhibited *ζ* values between +35–52 mV.

### Nanoprecipitation of amine-functional BDME-containing hyperbranched-polydendrons with varying primary chain structures

The *hyp*-PD structures comprising BDME as the branching agent were also subjected to nanoprecipitation at pH 7.8 and successfully formed nanoparticle dispersions (Table 4). Interestingly, the block copolymer G_2_-*p*(DEAEMA_17_-*b*-(*t*BuMA_33_-*co*-BDME_2.0_)) was able to form a stable nanoparticle dispersion despite the analogous EGDMA-containing G_2_-*p*(DEAEMA_17_-*b*-(*t*BuMA_33_-*co*-EGDMA_0.9_)) failing to do so at this pH; it is unclear why this is the case but the different chemical nature of BDME compared to EGDMA may impact the hydrophobicity of the branched copolymers, suggesting a fine balance of properties that is also seen in the EGDMA-derived block *hyp*-PDs of different *t*BuMA segment length (Table 4).

The nanoprecipitates formed from BDME-containing *hyp*-PDs have two direct comparisons to EGDMA-containing nanoparticles formed at pH 7.8, namely G_2_-*p*(DEAEMA_50_-*co*-EGDMA_0.9_) and G_2_-*p*(DEAEMA_33_-*co*-HPMA_17_-*co*-EGDMA_0.9_). The homo *hyp*-PD synthesised with BDME formed nanoparticles of very similar hydrodynamic diameter but broader PDI at near neutral pH (see ESI Fig. S24†); however, the statistical *hyp*-PD comprising HPMA and BDME formed larger nanoparticles with a relatively broad PDI (Table 4). Again, it is unclear why this is the case and a detailed study of this behaviour is required. Conversely, G_2_-*p*(DEAEMA_17_-*b*-(*t*BuMA_33_-*co*-BDME_2.0_)) formed nanoparticle dispersions of narrow PDI.

### Studying the pH response of nanoprecipitated *hyp*-PD particles

**Fig. 6:**
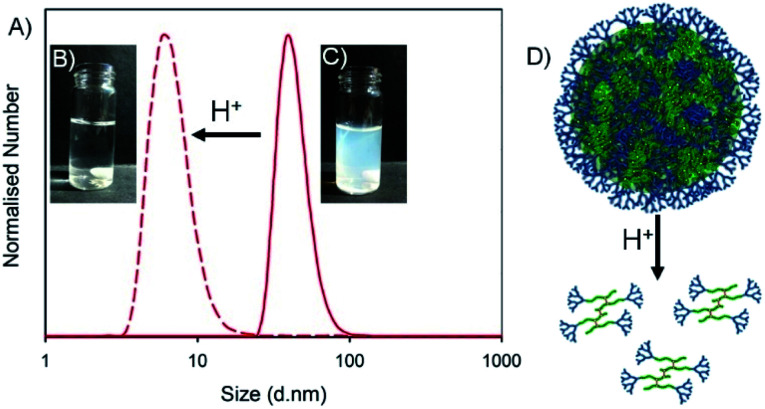
Example of pH-response for nanoprecipitates formed from G_2_-*p*(DEAEMA_50_-*co*-EGDMA_0.9_). (A) Impact of added HCl to aqueous dispersion of nanoparticles (*D*_n_ distribution shown); (B and C) visual impact of addition of HCl to the nanoprecipitate dispersion; (D) schematic representation of the disassembly of nanoprecipitates to component solvated hyperbranched-polydendrons.

The successful nanoprecipitates formed from this library of *hyp*-PDs were all generated in aqueous conditions that varied between pH 5.9–8.0 after solvent evaporation. Each of these nanoparticle dispersions were treated dropwise with aqueous HCl to form a final pH between 2.6–3.1 (Table 4). The visual turbidity from most samples was lost after treatment with HCl, indicating a swelling and disassembly of the nanoprecipitate dispersions (Fig. 6). DLS analysis of each sample showed a considerable loss of measured scattering intensity; for example, the nanoparticles formed from G_2_-*p*(DEAEMA_50_-*co*-EGDMA_0.9_) and the statistical *hyp*-PDs G_2_-*p*(DEAEMA_*x*_-*co*-HPMA_*y*_-*co*-EGDMA_0.9_) exhibited a decrease in derived count rate (DCR) to <5% of their initial value. Where sufficient but limited light scattering was present, the number distribution was observed to decrease to number average diameters (*D*_n_) of <10 nm (Fig. 6A, see ESI Fig. S25†). This behaviour was also seen for statistical *hyp*-PDs containing ≤50 mole % *t*BuMA within the primary chains of the branched macromolecules.

Interestingly, the *hyp*-PD derived nanoprecipitates formed from the statistical and block architectures containing 66 mole % *t*BuMA, namely G_2_-*p*(DEAEMA_17_-*co-t*BuMA_33_-*co*-EGDMA_0.9_), G_2_-*p*(DEAEMA_17_-*b*-(*t*BuMA_33_-*co*-EGDMA_0.9_)) and G_2_-*p*(DEAEMA_17_-*b*-(*t*BuMA_33_-*co*-BDME_2.0_)) showed an increase in *D*_z_ and *D*_n_ values after addition of HCl, suggesting the assemblies remain intact and swell during the protonation of the DEAEMA monomer residues (see ESI Fig. S26†).

These samples also represent the highest content of *t*BuMA in both block and statistical primary chain architectures and suggests a lack of penetration of aqueous acid into the hydrophobic core of the nanoprecipitate as this behaviour is clearly not evident when HPMA is present at high concentrations within the primary chains. Additionally, the hydrophobic environment within the block *hyp*-PD G_2-_*p*(DEAEMA_17_-*b*-(*t*BuMA_33_-*co*-BDME_2.0_))-derived nanoparticle acts to protect the acid sensitive brancher and avoid cleavage of the *hyp*-PD structure. The large variation in behaviour allows the judicious selection of monomers and copolymer compositions within the design of stimuli-responsive nanoprecipitates derived from *hyp*-PDs to target different responses under varying acidic conditions.

### Encapsulation, pH-triggered release, nanoparticle disassembly and *hyp*-polydendron degradation: dual response from a pH stimulus

**Fig. 7:**
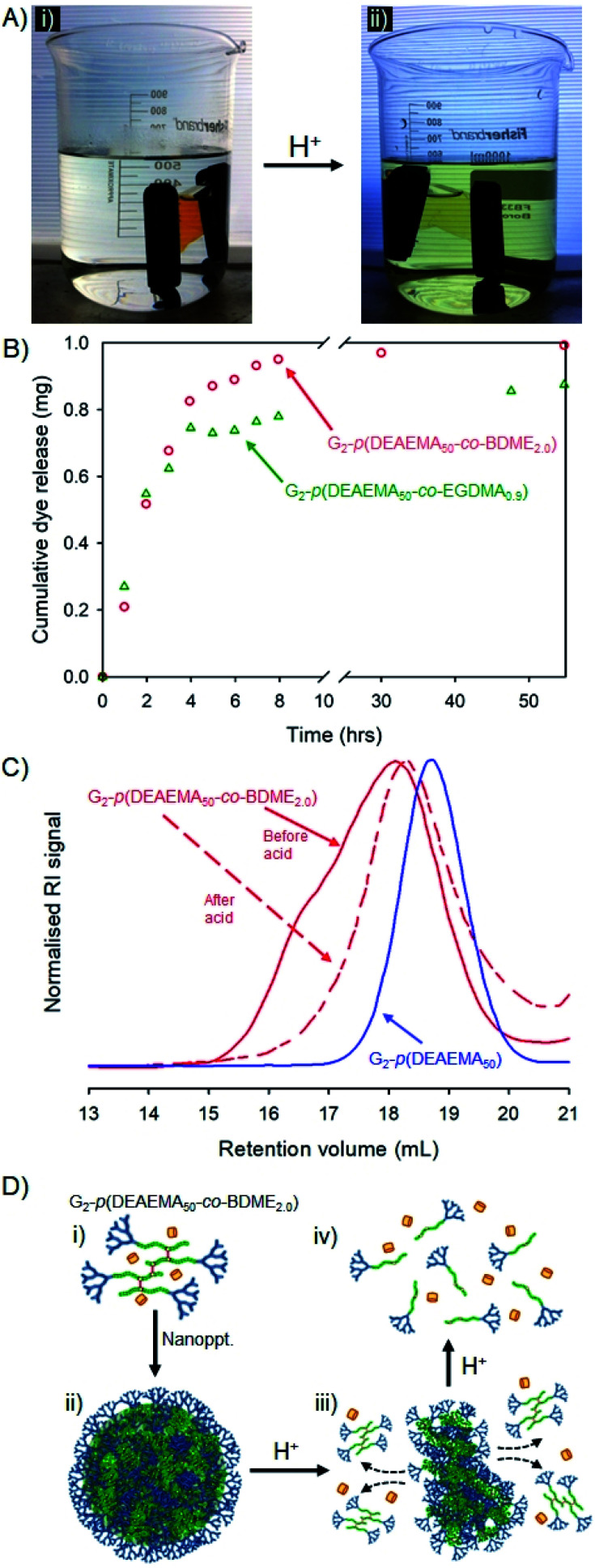
Stimulus-triggered response to addition of HCl to dye-loaded nanoprecipitates formed from G_2_-*p*(DEAEMA_50_-*co*-BDME_2.0_). (A(i)) Dye-loaded nanoprecipitates held within dialysis tubing in water; (A(ii)) visual triggering of dye release after HCl addition to reservoir water; (B) cumulative dye release and comparison to nanoprecipitates formed using EGDMA as brancher; (C) size exclusion chromatography showing considerable loss in molecular weight after HCl addition and comparison to primary chains; and (D) schematic representation of: (D(i)) solvated polymer and dye molecule, (D(ii)) formation of nanoprecipitate, (D(iii)) addition of acid and degradation of nanoprecipitate, and (D(iv)) degradation to primary chains.

The *hyp*-PD studies above provide a conceptual and experimental framework for the design of a guest-host nanoprecipitate that will function as a highly surface-functional carrier of a payload molecule, and allow for either pH-triggered disassembly and release or disassembly, release and degradation to low molecular weight polymer fragments (Fig. 7). As discussed above, this may have important implications in the environment or within *in vivo* settings.

Two materials, that vary solely in the brancher used, were selected for this demonstration, namely G_2_-*p*(DEAEMA_50_-*co*-EGDMA_0.9_) and G_2_-*p*(DEAEMA_50_-*co*-BDME_2.0_), and fluoresceinamine was selected as the guest molecule. Fluoresceinamine is a hydrophobic dye that is poorly water soluble, often used as a fluorescent tracer, and has good solubility in acetone. The two homo *hyp*-PDs were, therefore, individually dissolved in acetone (5 mg mL^−1^) and combined with an acetone solution of fluoresceinamine (1 mg mL^−1^) at a volume ratio allowing a dye-loading of 9 wt%. Each mixed acetone solution was nanoprecipitated into water (pH = 7.8) and yielded very similar orange/red, monomodal aqueous nanoparticle dispersions after evaporation of acetone (*D*_z_ = 45 or 40 nm and PDI = 0.197 or 0.190 for G_2_-*p*(DEAEMA_50_-*co*-EGDMA_0.9_) and G_2_-*p*(DEAEMA_50_-*co*-BDME_2.0_) respectively; see ESI Fig. S27†).

This slight decrease in hydrodynamic diameter relative to ‘blank’ nanoprecipitates implies a role for the hydrophobic fluorescer in modifying the early stages of the nanoprecipitation process. The smaller *D*_z_ value for the guest/host nanoprecipitate formed from G_2_-*p*(DEAEMA_50_-*co*-BDME_2.0_) may indicate the presence of a small number of methacrylic acid residues within the *hyp*-PD structure. These may have formed by undesired cleavage of the BDME acetal prior to nanoprecipitation and induce a fluorescer/polymer interaction that facilitates the particle formation. NMR studies were unable to identify carboxylic acid functionality at the possible low concentrations due to considerable resonance overlap.

The dye-loaded aqueous dispersions were transferred to dialysis bags (molecular weight cut-off = 2000 g mol^−1^) and left to stand in pure water for 48 hours after which the reservoir water of each sample showed a slight yellow colour that was quantified by UV-visible spectroscopy against a standard curve (ESI Fig. S28†). The encapsulation efficiency of each *hyp*-polydendron nanoprecipitate was, therefore, calculated as 74% and 99% for G_2_-*p*(DEAEMA_50_-*co*-EGDMA_0.9_) and G_2_-*p*(DEAEMA_50_-*co*-BDME_2.0_) respectively, suggesting again that the presence of BDME modifies the internal environment of the nanoparticles. The samples were transferred to fresh water and left to stand at ambient temperature for a further 24 hours after which the water remained clear and uncoloured (Fig. 7A(i)).

The addition of HCl to the reservoir water, to achieve a pH of approximately 2, led to an observable yellow colour leaching from the dialysis tubing within 30 minutes (Fig. 7A(ii)) which was monitored and quantified by UV-visible spectroscopy over a 60 hour period (Fig. 7B).

Although both *hyp*-PD nanoprecipitates demonstrated a pH-responsive release, the release from the BDME-containing nanoprecipitates was rapid and led to a greater cumulative mass of released fluorescer; this is interesting to note as the presence of BDME within the nanoparticle clearly led to a higher encapsulation efficiency, suggesting a more favourable environment within the nanoparticle. The addition of HCl would be expected to swell the nanoparticles formed from G_2-_*p*(DEAEMA_50_-*co*-EGDMA_0.9_) or G_2_-*p*(DEAEMA_50_-*co*-BDME_2.0_), leading to dissolution and disassembly, but in the case of G_2_-*p*(DEAEMA_50_-*co*-BDME_2.0_) an additional degradation of the linking BDME chemistry would be expected to further break down the protonated macromolecules into their respective LDH primary chains, as seen in acetone solution studies (Fig. 4).

In order to study the impact of the acid trigger on the molecular weight distribution of the *hyp*-PDs within the nanoprecipitates, the contents of the dialysis tubing were removed, dried and reprecipitated from THF solution into hexane, prior to filtration and drying *in vacuo* overnight. Despite the very low mass of the recovered samples, SEC analysis was possible (Fig. 7C and Table 5) and comparison with the corresponding LDH and *hyp*-PD was undertaken.

**Table 5:** Size exclusion chromatography analysis of homo hyperbranched-polydendron nanoprecipitates and polymers synthesised using either BDME or EGDMA before and after treatment with HCl and subsequent purification

*hyp*-PD polymer & acid treatment	TD-SEC^a^		*Ð*
	*M*_n_ (g mol^−1^)	*M*_w_ (gmol^−1^)	
G_2_-*p*(DEAEMA_50_-*co*-BDME_2.0_)	157 300	321 100	2.04
After acid addition (particle in water)	68 800	99 500	1.45
After acid addition (solution in acetone)	39 950	51 150	1.28
G_2_-*p*(DEAEMA_50_-*co*-EGDMA_0.9_)	125 700	341 800	2.72
After acid addition (particle in water)	1 513 000	3 938 000	2.60
After acid addition (solution in acetone)	—	—	—

a Triple detection size exclusion chromatography using THF/2% TEA eluent; all.

The nanoparticle dispersion generated from G_2_-*p*(DEAEMA_50_-*co*-BDME_2.0_) showed a considerable decrease in molecular weight after triggered release using HCl, as would be expected from previous studies of fully dissolved *hyp*-PD in acetone. Noticeably, TD-SEC analysis does not show a complete degradation to primary LDH chains suggesting incomplete degradation; however, the molecular weight distribution overlays well with the corresponding G_2_-*p*(DEAEMA_50_) LDH (Fig. 7C) and the process of acid-degradation within the dialysis tubing and subsequent reprecipitation may well have led to removal of part of the low molecular weight fraction within the sample. In stark contrast, purification and analysis of the G_2_-*p*(DEAEMA_50_-*co*-EGDMA_0.9_) derived sample led to a considerable *increase* in observed molecular weight (see ESI Fig. S29†), also suggesting a removal of a portion of the lower molecular weight species and caution must be exercised with the characterisation of this sample. As mentioned earlier, the potential to encapsulate and release guest molecules, with subsequent triggered nanoparticle degradation has potential widespread application.

## Conclusions

The hyperbranched-polydendron platform offers considerable flexibility to design complex macromolecules that are able to form uniform nanoparticles under the relatively facile process of rapid nanoprecipitation. The primary chains of the *hyp*-PDs may be varied considerably, allowing the tuning of behaviour that translates to control of the resulting nanoparticles including their response to nanoprecipitation and subsequent stimuli.

Importantly, the balance of the branched *hyp*-PD structure, which is dominated by covalent bonding and chemical functionality, and the nanoprecipitation process, which is dominated by non-covalent self-assembly, may be subject to considerable tuning. Within the current study, the pH-response within aqueous nanoparticle dispersions was dictated by the design of the branched polymer building blocks with options ranging from dissolution to the constituent branched building blocks, simple swelling after protonation of tertiary amine functionality, or a combination of swelling, dissolution and branched polymer degradation to yield a solution of linear-dendritic hybrid primary chain components.

The versatility of this approach to designing nanoparticle behaviour through branched polymer chemistry and architecture offers considerable opportunities within future applications, including environment specific degradation routes such as enzymatic mechanisms.^38^ The range of dendron initiators that may be synthesised and combined with commercially available monomers and divinyl branchers is substantial and the ease of synthesis of these complex materials presents a facile route to new material study and optimisation.

## Conflicts of interest

HER, PC, FYH, AO and SPR are coinventors of patented inventions related to hyperbranched-polydendrons.

## Supplementary Material

NA-002-D0NA00696C-s001
