# Supplementary material for: Designing single trigger/dual-response release and degradation into amine-functional hyperbranched-polydendron nanoprecipitates
Source: Nanoscale Adv. 2020 Oct 28;2(11):5468–77. doi: 10.1039/d0na00696c (PMC9418457; doi:10.1039/d0na00696c)
Supplement: NA-002-D0NA00696C-s001 [file NA-002-D0NA00696C-s001.pdf]

Electronic Supplementary Material (ESI) for Nanoscale Advances.
This journal is © The Royal Society of Chemistry 2020

Electronic Supplementary Information

Designing single trigger/dual-response release and degradation into amine-functional hyperbranched-polydendron nanoprecipitates.

Hannah E. Rogers, Pierre Chambon, Sean Flynn, Faye Y. Hern, Andrew Owen and Steve P Rannard

Materials and suppliers

2-Hydroxypropyl methacrylate (HPMA, 97% mixture of isomers), 2-(diethyl amino)ethyl methacrylate (DEAEMA, 99%), tert-butyl methacrylate (*t*BuMA, 98%), ethylene glycol dimethacrylate (EGDMA, 98%), methacrylic acid (MAA, 99 %), copper(I)chloride (Cu(I)Cl, ≥99%), 2,2'-bipyridyl (bpy, ≥ 99%), 1, 4-butanediol divinyl ether (99 %), 4-tert-butylcatechol (≥ 99 %),fluoresceinamine (isomer I), triethylamine (TEA, 99%), and basic aluminium oxide were purchased from Sigma Aldrich and used without any further purification. All solvents were analytical grade and purchased from Fisher scientific and used as received.

Analysis and analytical Equipment

Analytical thin layer chromatography (TLC) was performed on commercial Merck Plates coated with silica gel. Nuclear Magnetic Resonance (NMR) spectroscopy spectra were collected using a Bruker DPX-400 spectrometer. ^{1}H NMR spectra were recorded at 400 MHz and ^{13}C NMR spectra were recorded at 100 MHz. Deuterated chloroform ($CDCl_3$) and deuterated methanol (CD_3OD) purchased from Sigma Aldrich were used as NMR solvents. Chemical shifts (δ) are reported in parts per million (ppm) and TMS was used as an internal standard for both ^{1}H and ^{13}C spectra. Electrospray mass spectrometry data (ES-MS) was recorded in the Mass Spectrometry Laboratory at the University of Liverpool. The data was obtained using a MicroMass LCT mass spectrometer using electron ionisation and direct infusion syringe pump sampling. All materials were diluted with methanol. Elemental analyses were obtained from a Thermo FlashEA 1112 series CHNSO elemental analyser. Size Exclusion Chromatography (SEC) was carried out using a Malvern Viscotek GPC Max connected to a Viscotek 270 Light Scattering detector, Viscometer and Refractive index (RI) triple detection system. HPLC grade THF (Fisher) containing 2% TEA (Sigma Aldrich) was used as the eluent, with a flow rate of 1 mL/min. SEC Columns were mixed bed columns supplied by Viscotek. The column oven was set at 35°C. The obtained chromatograms were analysed using Malvern OmniSec software calibrated by a universal calibration calculation relative to a narrow linear polystyrene standard (105 kg/mol). Nanoparticle characterisation were carried out on a Malvern Zetasizer Nano ZS dynamic light scattering (DLS) instrument. Ultraviolet-visible spectrophotometry was conducted using a Thermo Scientific NanoDrop 2000c spectrometer monitoring the absorption at λ_{max} = 496 nm operating in cuvette mode.

Experimental procedures

The synthesis of the second-generation amine functional dendron initiator, **1**, was accomplished using previously reported procedures[1]

Synthesis of 'homo' linear dendritic hybrids exemplified by G_2-*p*(*t*BuMA$_{50}$) – In a typical synthesis, bpy (134.9 mg, 0.8637 mmol, 2 eq.), *t*BuMA (3.07 g, 21.59 mmol, 50 eq.) and IPA/H_2O (92.5:7.5 %; 39 wt% based on monomer) were placed into a 25 mL RBF. The solution was stirred and deoxygenated using a N_2 purge for 15 minutes. Cu(I)Cl (42.8 mg, 0.4318 mmol, 1 eq.) was added to the flask and left to purge for a further 5 minutes. The second-generation dendron initiator, **1**, (0.3934 g, 0.4318 mmol, 1 eq.) was added to the flask under a positive flow of N_2 and the solution was left to polymerise at 40°C. Reactions were terminated when >99 % conversion was reached, as judged by ^{1}H NMR, by exposure to oxygen and addition of THF. The catalyst residues were removed by passing the mixture over a basic alumina column. THF was removed under vacuum to concentrate the sample before precipitation into cold hexane.

Synthesis of 'statistical' linear dendritic hybrids exemplified by G_2-*p*(DEAEMA$_{25}$-*co*-HPMA$_{25}$) – In a typical synthesis, bpy (134.9 mg, 0.8637 mmol, 2 eq.), DEAEMA (2 g, 10.80 mmol, 25 eq.), HPMA (1.5567 g, 10.80 mmol, 25 eq.) and IPA/H_2O (92.5:7.5 %; 39 wt% based on monomer) were placed into a 25 mL RBF. The solution was stirred and deoxygenated using a N_2 purge for 15 minutes. Cu(I)Cl (42.8 mg, 0.4318 mmol, 1 eq.) was added to the flask and left to purge for a further 5 minutes. The second-generation dendron initiator, **1**, (0.3934 g, 0.4318 mmol, 1 eq.) was added to the flask under a positive flow of N_2, and the solution was left to polymerise at 40°C. Reactions were terminated when >99 % conversion was reached, as judged by ^{1}H NMR, by exposure to oxygen and addition of acetone. The catalyst residues were removed by passing the mixture over a basic alumina column. Acetone was removed under vacuum to concentrate the sample before precipitation into cold petroleum ether (40–60).

Synthesis of 'block' linear dendritic hybrids exemplified by G_2-*p*(DEAEMA$_{25}$-*b*-*t*BuMA$_{25}$) – In a typical synthesis, bpy (134.9 mg, 0.8637 mmol, 2 eq.), DEAEMA (2.00 g, 10.80 mmol, 25 eq.) and IPA/H_2O (92.5:7.5 %; 39 wt% based on monomer) were placed into a 25 mL RBF. The solution was stirred and deoxygenated using a N_2 purge for 15 minutes. Cu(I)Cl (42.8 mg, 0.4318 mmol, 1 eq.) was added to the flask and left to purge for a further 5 minutes. The second-generation dendron initiator, **1**, (0.3934 g, 0.4318 mmol, 1 eq.) was added to the flask under a positive flow of N_2, and the solution was left to polymerise at 40°C. At a measured >80 % monomer conversion, *t*BuMA (1.54 g, 10.80 mmol, 25 eq.) was introduced in IPA/H_2O (92.5:7.5 %; 39 wt% based on monomer). Reactions were terminated when >99 % conversion was reached, as judged by ^{1}H NMR, by exposure to oxygen and addition of

acetone. The catalyst residues were removed by passing the mixture over a basic alumina column. Acetone was removed under vacuum to concentrate the sample before precipitation into cold petroleum ether (40–60).

Synthesis of 'homo' hyperbranched polydendron exemplified by G_2-*p*($DEAEMA_{50}$-*co*-$EGDMA_{0.9}$) – In a typical synthesis, bpy (134.9 mg, 0.8637 mmol, 2 eq.), DEAEMA (4 g, 21.59 mmol, 50 eq.), EGDMA (77.0 mg, 0.3886 mmol, 0.9 eq.) and IPA/H_2O (92.5:7.5 %; 39 wt% based on monomer) were placed into a 25 mL RBF. The solution was stirred and deoxygenated using a N_2 purge for 15 minutes. Cu(I)Cl (42.8 mg, 0.4318 mmol, 1 eq.) was added to the flask and left to purge for a further 5 minutes. The second-generation dendron initiator, **1**, (0.3934 g, 0.4318 mmol, 1 eq.) was added to the flask under a positive flow of N_2, and the solution was left to polymerise at 40°C. Reactions were terminated when >99 % conversion was reached, as judged by ^{1}H NMR, by exposure to oxygen and addition of acetone. The catalyst residues were removed by passing the mixture over a basic alumina column. Acetone was removed under vacuum to concentrate the sample before precipitation into cold petroleum ether (40-60).

Synthesis of 'statistical' hyperbranched polydendron exemplified by G_2-*p*($DEAEMA_{25}$-*co*-*t*$BuMA_{25}$-*co*-$EGDMA_{0.9}$) – In a typical synthesis, bpy (134.9 mg, 0.8637 mmol, 2 eq.), DEAEMA (2 g, 10.80 mmol, 25 eq.), *t*BuMA (1.5352 g, 10.80 mmol, 25 eq.), EGDMA (77.0 mg, 0.3886 mmol, 0.9 eq.) and IPA/H_2O (92.5:7.5 %; 39 wt% based on monomer) were placed into a 25 mL RBF. The solution was stirred and deoxygenated using a N_2 purge for 15 minutes. Cu(I)Cl (42.8 mg, 0.4318 mmol, 1 eq.) was added to the flask and left to purge for a further 5 minutes. The second-generation dendron initiator, **1**, (0.3934 g, 0.4318 mmol, 1 eq.) was added to the flask under a positive flow of N_2, and the solution was left to polymerise at 40°C. Reactions were terminated when >99 % conversion was reached, as judged by ^{1}H NMR, by exposure to oxygen and addition of acetone. The catalyst residues were removed by passing the mixture over a basic alumina column. Acetone was removed under vacuum to concentrate the sample before precipitation into cold petroleum ether (40–60).

Synthesis of 'block' hyperbranched polydendron exemplified by G_2-*p*($DEAEMA_{25}$-*b*-(*t*$BuMA_{25}$-*co*-$EGDMA_{0.9}$)) – In a typical synthesis, bpy (134.9 mg, 0.8637 mmol, 2 eq.), DEAEMA (2.00 g, 10.80 mmol, 25 eq.) and IPA/H_2O (92.5:7.5 %; 39 wt% based on monomer) were placed into a 25 mL RBF. The solution was stirred and deoxygenated using a N_2 purge for 15 minutes. Cu(I)Cl (42.8 mg, 0.4318 mmol, 1 eq.) was added to the flask and left to purge for a further 5 minutes. The second-generation dendron initiator, **1**, (0.3934 g, 0.4318 mmol, 1 eq.) was added to the flask under a positive flow of N_2, and the solution was left to polymerise at 40°C. At a measured >80 % monomer conversion, *t*BuMA (1.54 g, 10.80 mmol, 25 eq.) and EGDMA (77 mg, 0.389 mol, 0.9 eq.) was introduced. Reactions were terminated when >99 % conversion was reached, as judged by ^{1}H NMR, by exposure to oxygen and addition of acetone. The catalyst residues were removed by passing the mixture over a basic alumina

column. Acetone was removed under vacuum to concentrate the sample before precipitation into cold petroleum ether (40–60).

Synthesis of 1,4-butanediol di(methacryoyloxy)-ethyl ether (BDME, 6) – minor modification of previously reported synthesis[2]

1, 4-Butanediol divinyl ether (5.03 g, 5.6 mL, 35.36 mmol, 1 eq.) was added to a two-necked 250 mL RBF equipped with a condenser, a magnetic stirrer and a positive flow of N_2. A small amount of radical inhibitor 4-*tert*-butylcatechol (approx. 5mg) was added and the mixture deoxygenated using a N_2 purge for 15 minutes. Once dissolved, the temperature was raised to 70ºC. MAA (15.22 g, 15.1 mL, 0.1769 mol, 5 eq.) was added drop wise over 10 minutes through a septum. The reaction was allowed to proceed at 70ºC for a further 6 hours with stirring. After this time, the reaction was stopped by cooling and exposing to the air. The crude product was dissolved in chloroform (100 mL) and washed with base (KOH aq, 0.02 M, 3 x 100 mL). The combined washings were collected and dried over $NaSO_4$ and the solvent removed by rotary evaporation. Yield: 8.73 g, yellow oil, (79%). ^{1}H NMR (400 MHz, $CDCl_3$) δ 1.43 (d, 6H), 1.65 (m, 4H), 1.95 (s, 6H), 3.51 (m, 2H), 3.68 (m, 2H), 5.60 (s, 2H), 5.97 (m, 2H), 6.15 (s, 2H). ^{13}C NMR (100 MHz, CDCl3) δ 18.4, 20.6, 26.0, 30.9, 68.9, 97.0, 126.0, 136.5, 166.9. Calcd $[M+H]^+$ ($C_{16}H_{26}O_6$) m/z = 314.4. Found: ESI-MS: $[M+Na]^+$ m/z = 337.2 and [M+K]+ m/z = 353.1. Anal. Calcd for $C_{16}H_{26}O_6$: C, 61.07; H, 8.27%. Found C, 61.33; H, 8.45%.

Typical procedure for aqueous nanoprecipitation – The LDH and *hyp*-PD materials were dissolved in acetone at a concentration of 5 mg mL^{-1}. 2 mL of this solution was subjected to a rapid solvent switch through addition into 10 mL of water, to give a final polymer nanoparticle concentration of 1 mg mL^{-1} in water after acetone removal by evaporation overnight.

Typical procedure for fluoresceinamine encapsulation – FA was dissolved in acetone at a concentration of 1 mg mL^{-1}. 1 mL of this solution, along with 2 mL of the *hyp*-PD solution (5 mg mL^{-1}), was subjected to a rapid solvent switch through addition into 10 mL of water, to give a final polymer nanoparticle concentration of 1 mg mL^{-1} in water after acetone removal by evaporation overnight.

Typical procedure for solution hydrolysis of BDME-containing *hyp*-PDs – The *hyp*-PD was dissolved in acetone at a concentration of 40 mg mL^{-1} (9 mL). HCl (5M, 400 μL) was added drop wise to the solution and stirred vigorously at room temperature for 20 minutes, resulting in a cloudy solution with solid precipitate. Deionised water (9 mL) was added to the acidic polymer solution and left to stir overnight in a sealed vial. The hydrolysed polymer solution was frozen in liquid nitrogen and lyophilized for 72 hours.

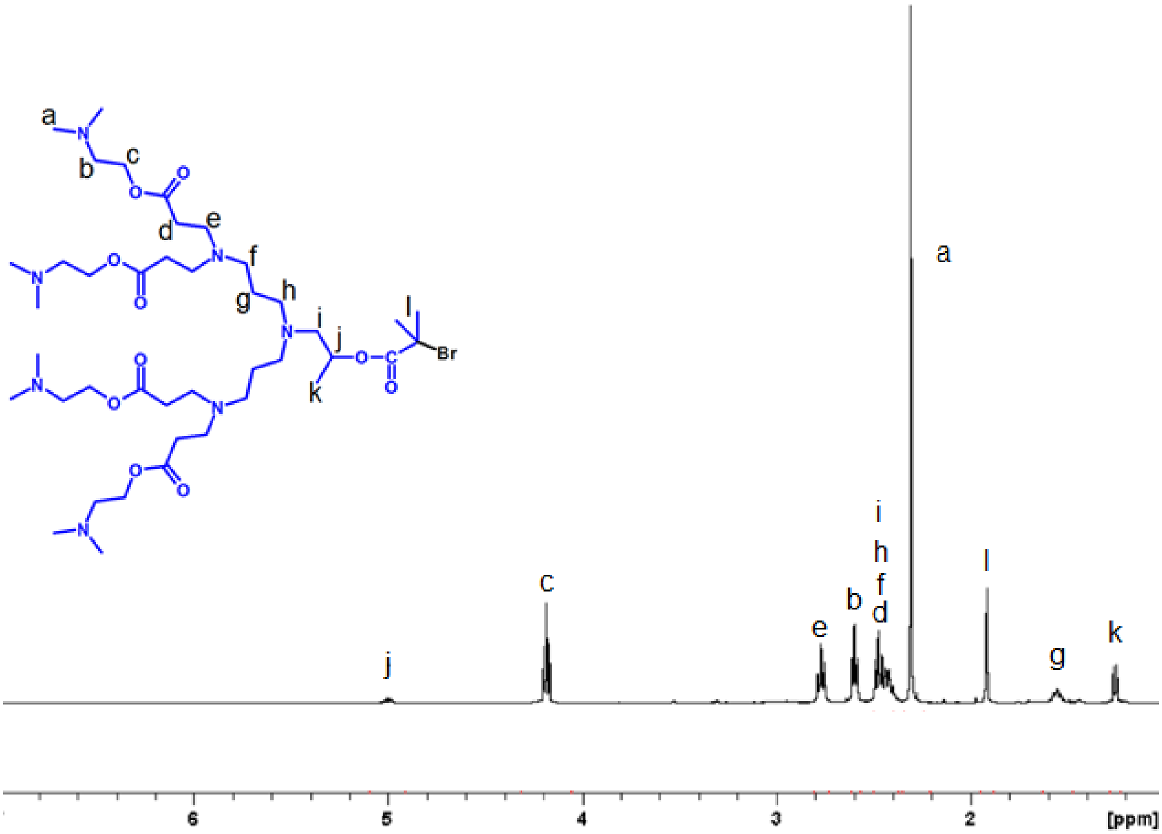

Figure S1. ^{1}H NMR spectrum ($CDCl_3$, 400 MHz) spectrum of G2 initiator, **1.**

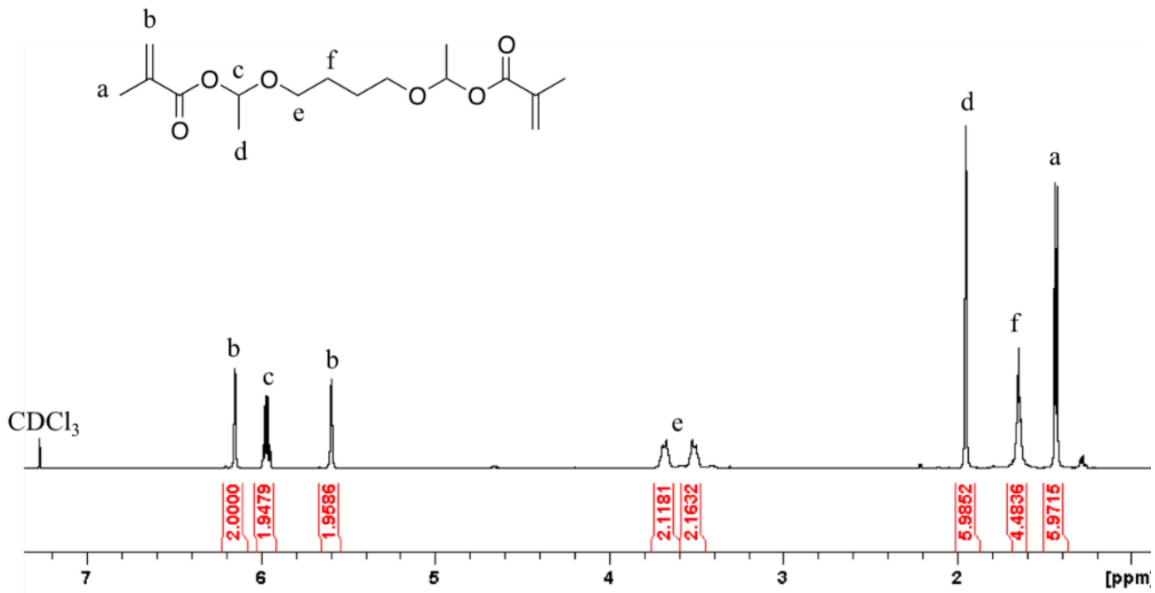

Figure S2. ^{1}H NMR ($CDCl_3$, 400 MHz) spectrum of BDME brancher

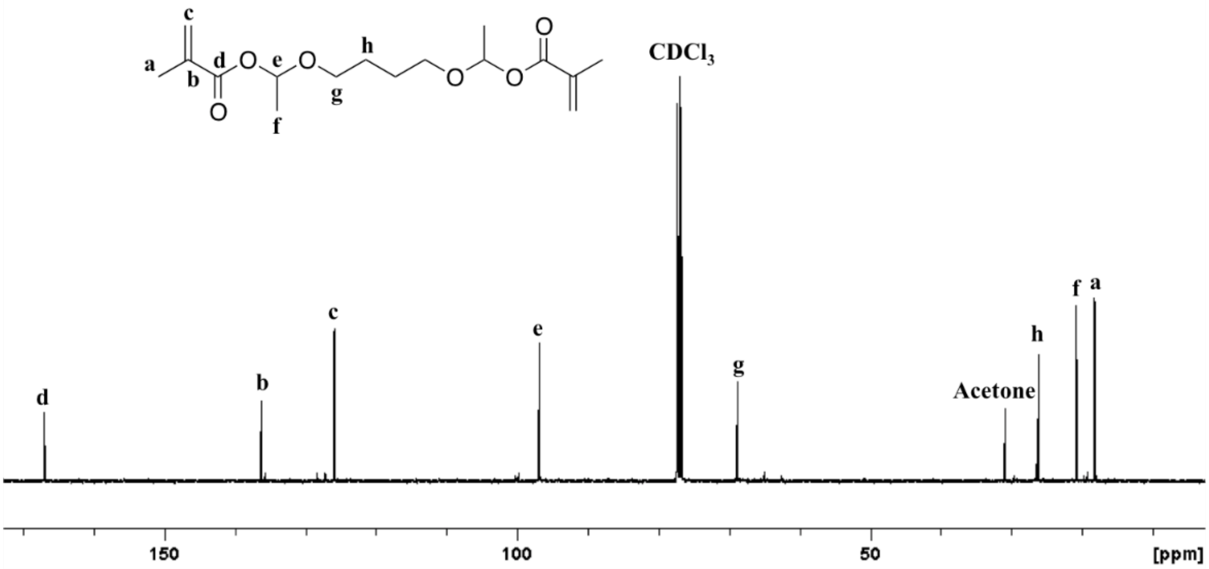

Figure S3. ^{13}C NMR ($CDCl_3$, 100 MHz) spectrum of BDME brancher

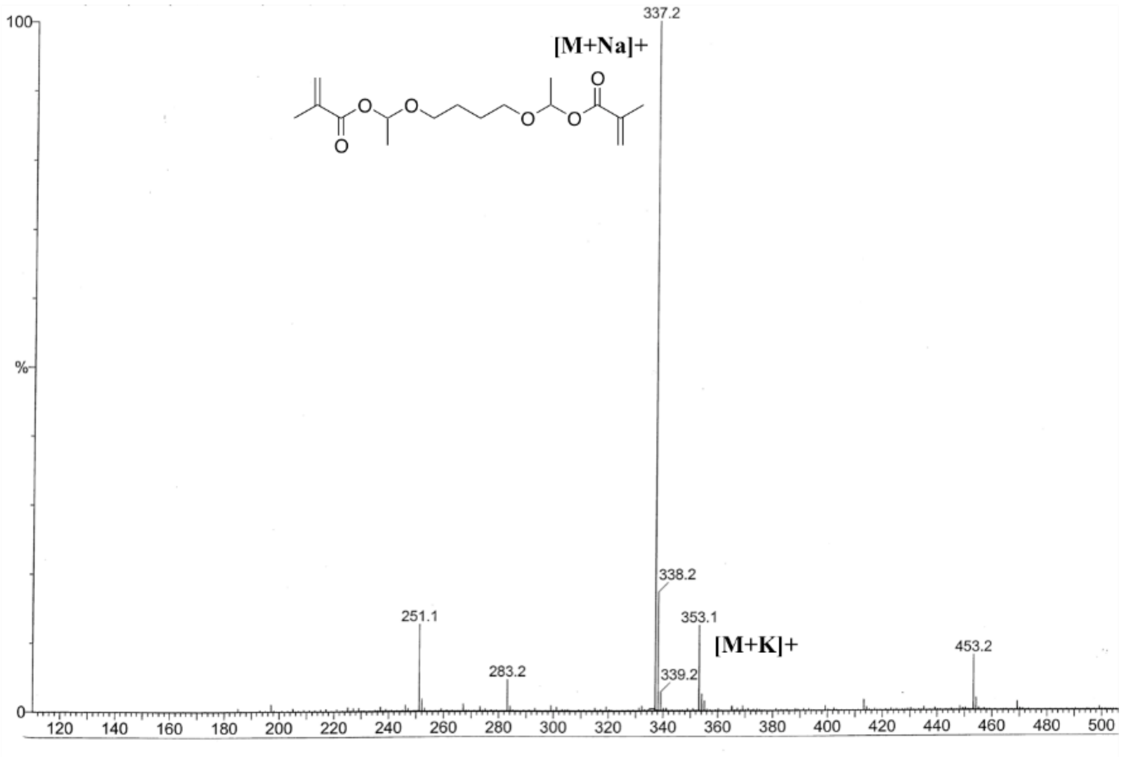

Figure S4. Electrospray mass spectrum of BDME brancher

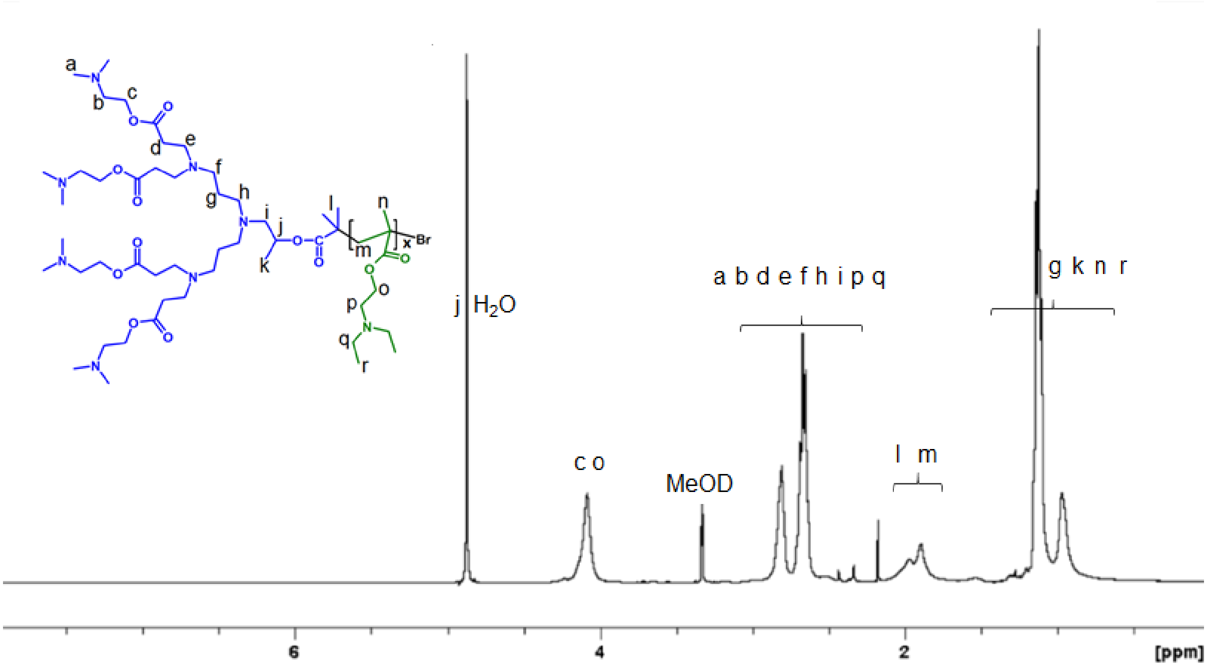

Figure S5. ^{1}H NMR spectrum (MeOD, 400 MHz) of G_2-*p*($DEAEMA_{50}$) (homo LDH)

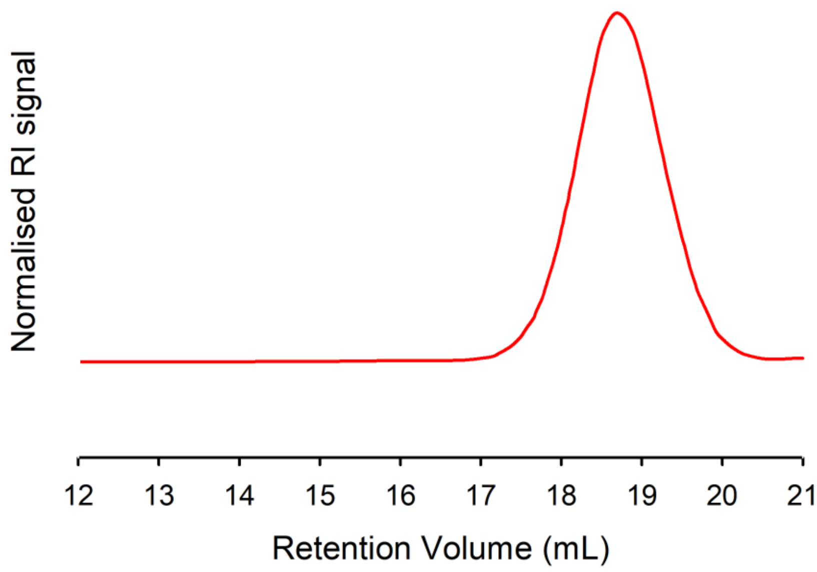

Figure S6. Triple detection size exclusion chromatography (RI chromatogram; THF/2% TEA eluent) of G_2-*p*($DEAEMA_{50}$) (homo LDH)

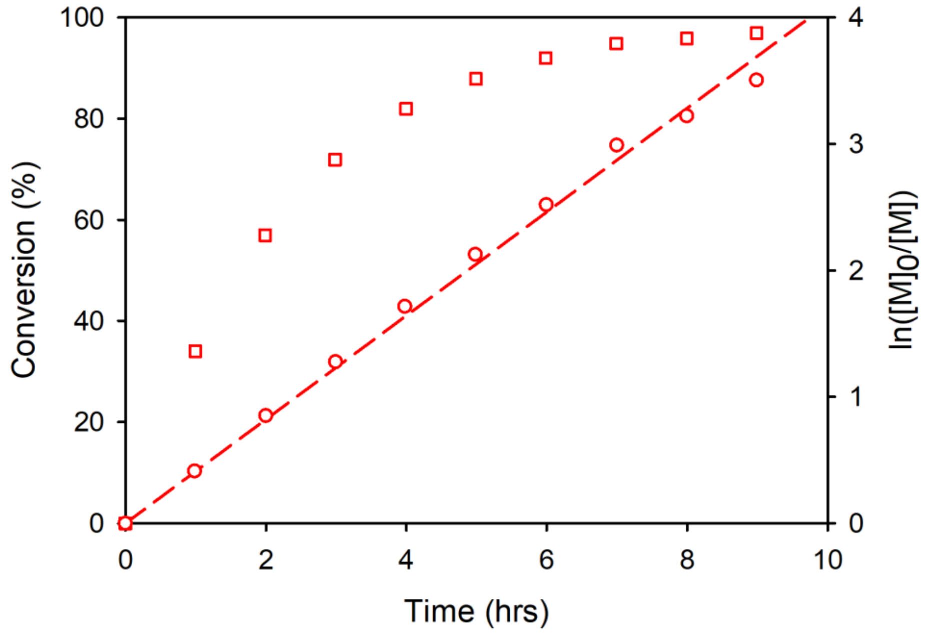

Figure S7. Kinetic analysis of the ATRP of G_2-*p*($DEAEMA_{50}$) (homo LDH). Open squares = conversion *vs.* time; open circles = semi-logarithm *vs.* time.

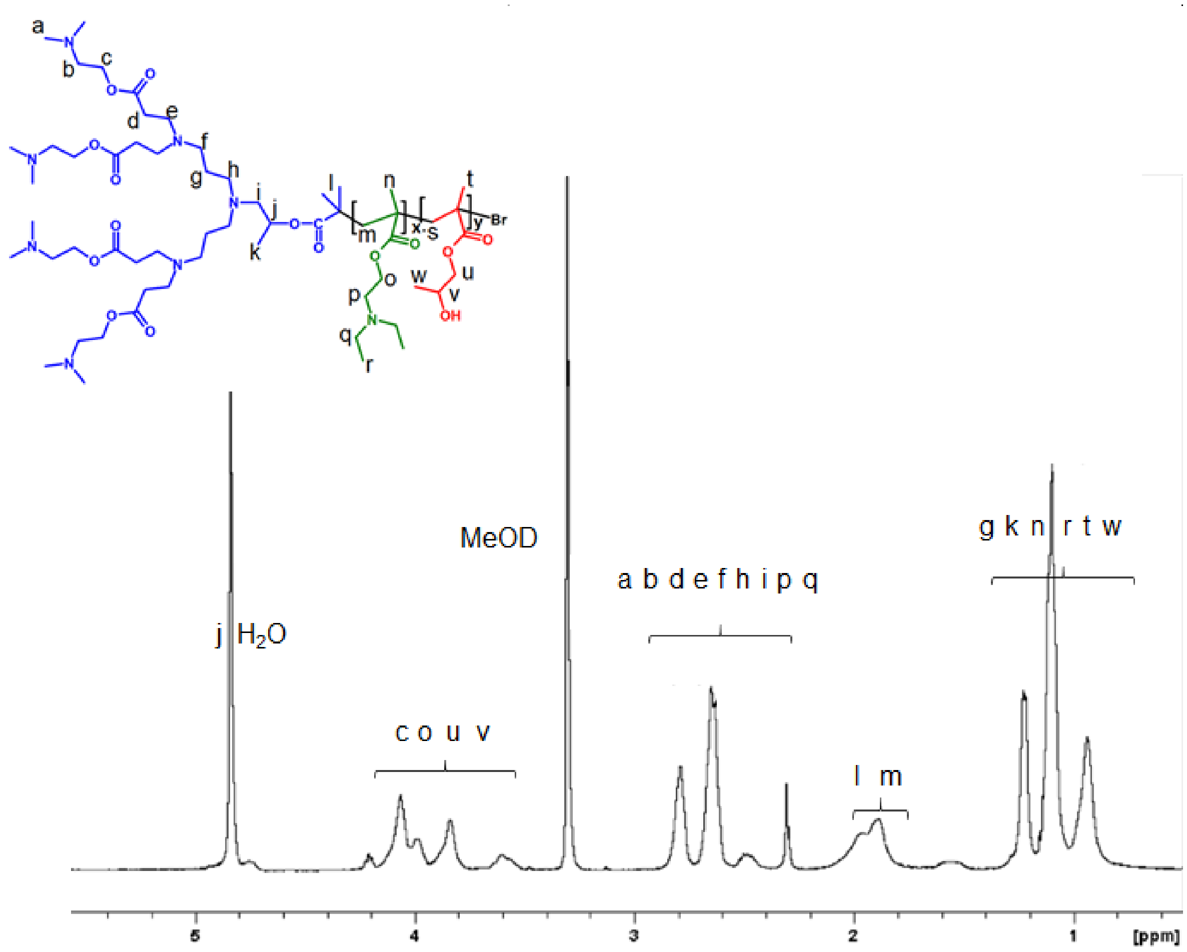

Figure S8. ^{1}H NMR spectrum (CD_3OD, 400 MHz) of G_2-*p*($DEAEMA_{33}$-*co*-$HPMA_{17}$) (statistical LDH)

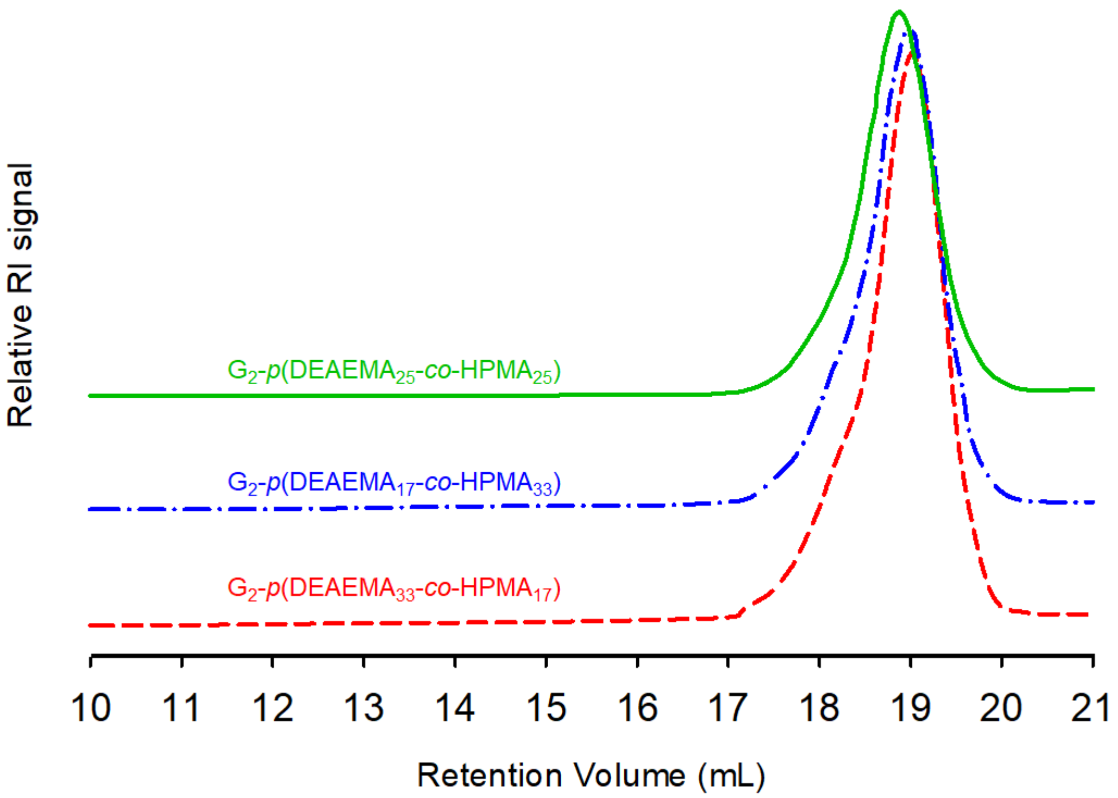

Figure S9. Triple detection size exclusion chromatography (overlaid RI chromatograms; THF/2% TEA eluent) of G_2-*p*($DEAEMA_y$-*co*-$HPMA_x$) (statistical LDH)

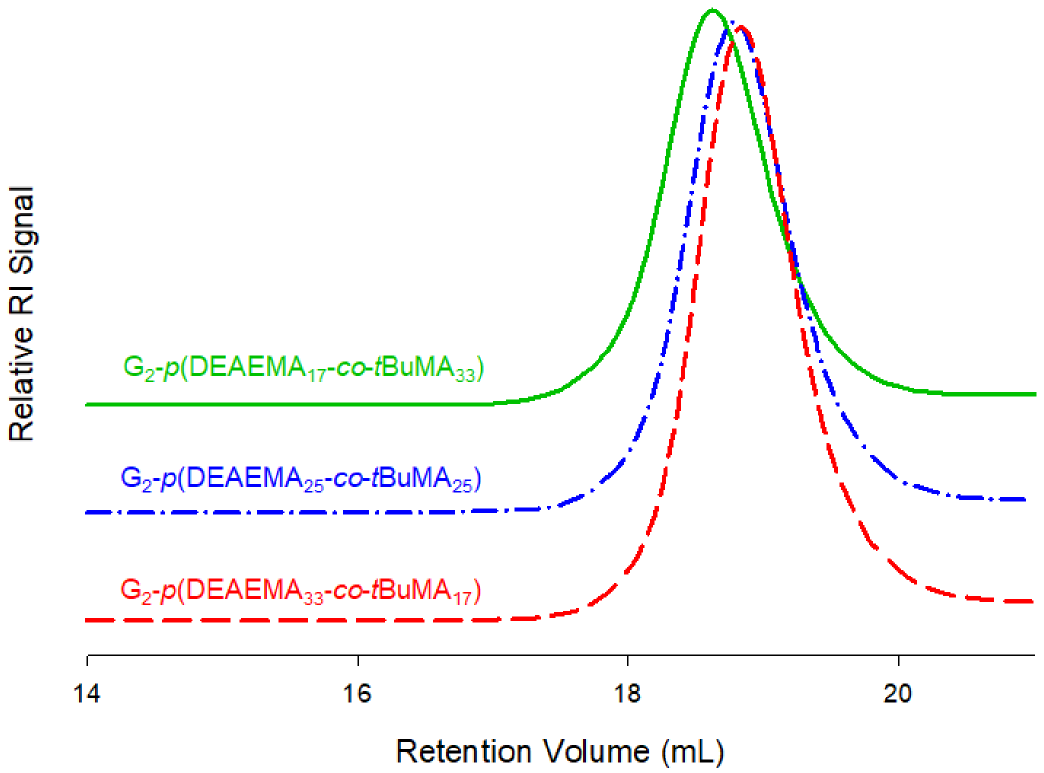

Figure S10. Triple detection size exclusion chromatography (overlaid RI chromatograms; THF/2% TEA eluent) of G_2-*p*($DEAEMA_y$-*co*-*t*$BuMA_x$) (statistical LDH)

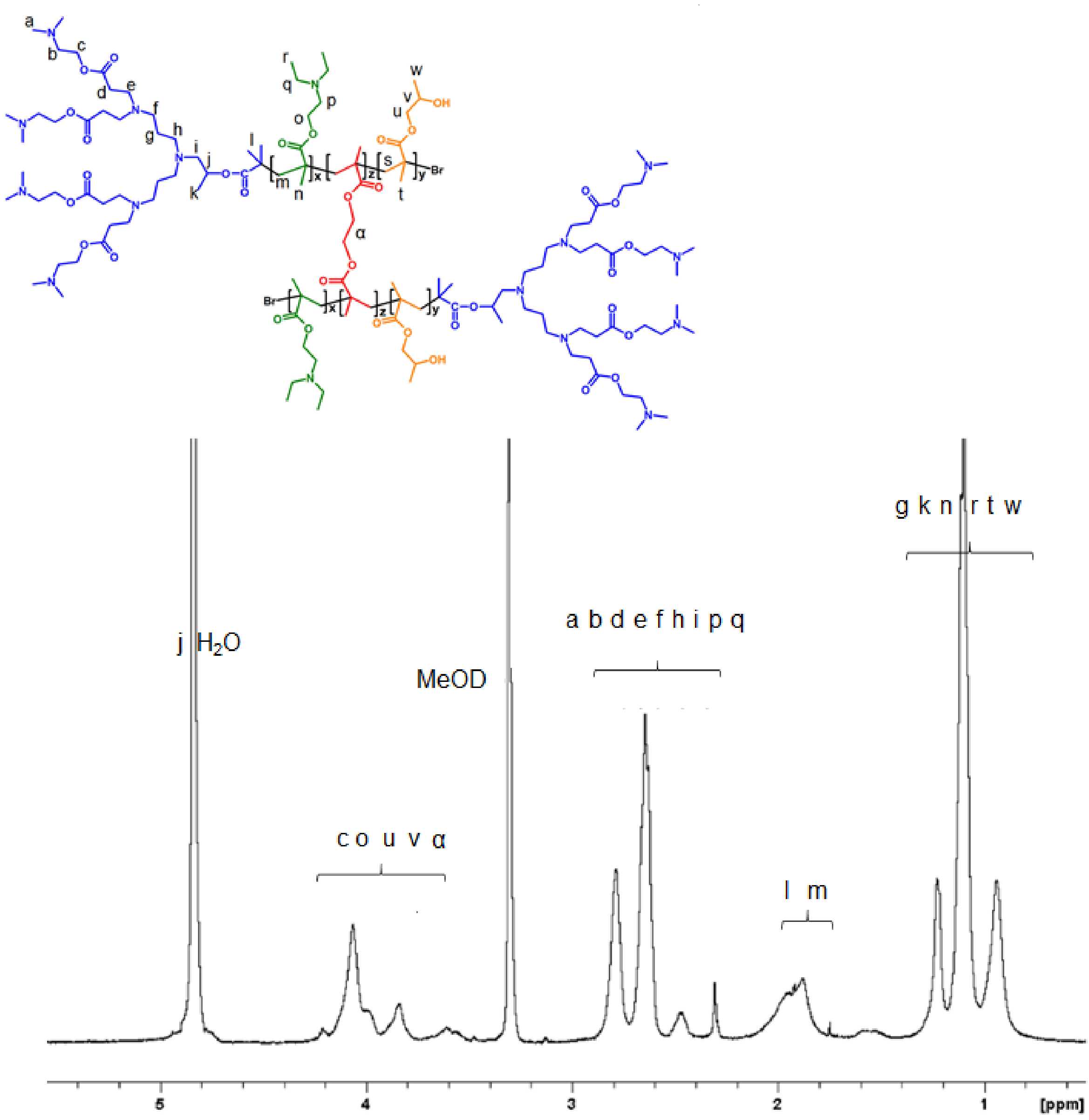

Figure S11. ^{1}H NMR spectrum (CD_3OD, 400 MHz) of G_2-*p*(DEAEMA$_{33}$-*co*-HPMA$_{17}$ -*co*-EGDMA$_{0.9}$) (statistical *hyp*-PD)

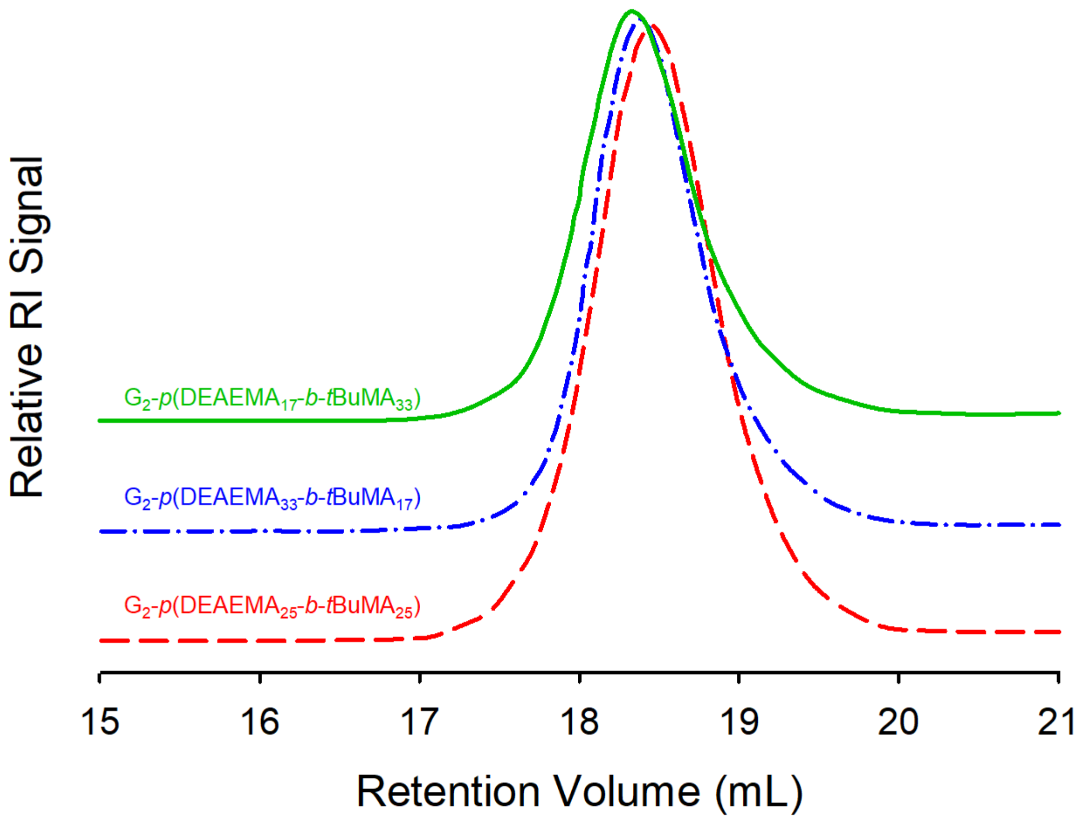

Figure S12. Triple detection size exclusion chromatography (overlaid RI chromatograms; THF/2% TEA eluent) of G_2-*p*(DEAEMA$_y$-*b*-*t*BuMA$_x$) (block LDH)

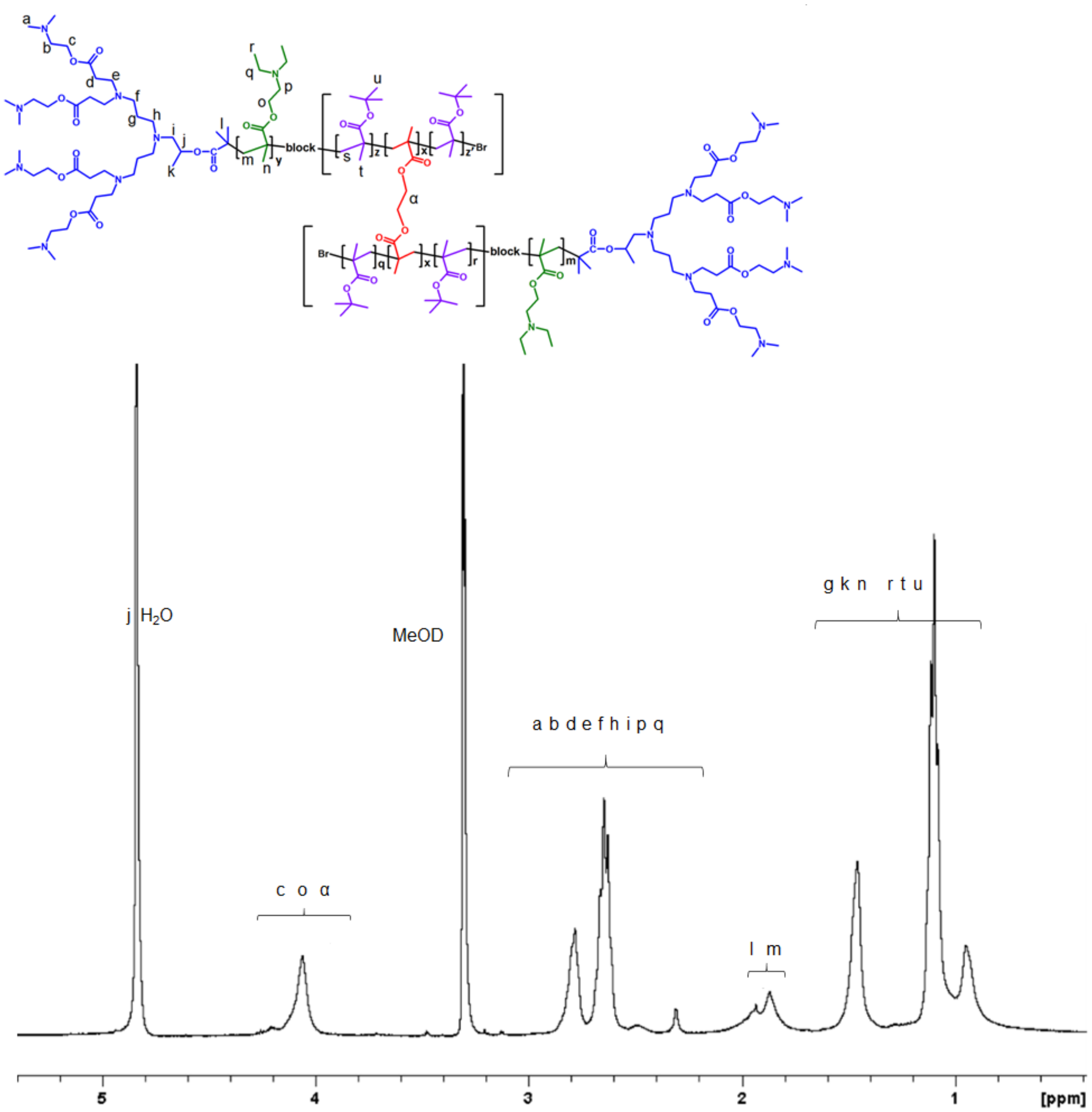

Figure S13. ^{1}H NMR spectrum (CD_3OD, 400 MHz) of G_2-*p*($DEAEMA_{25}$-*b*-(*t*$BuMA_{25}$-*co*-$EGDMA_{0.9}$)) (block *hyp*-PD)

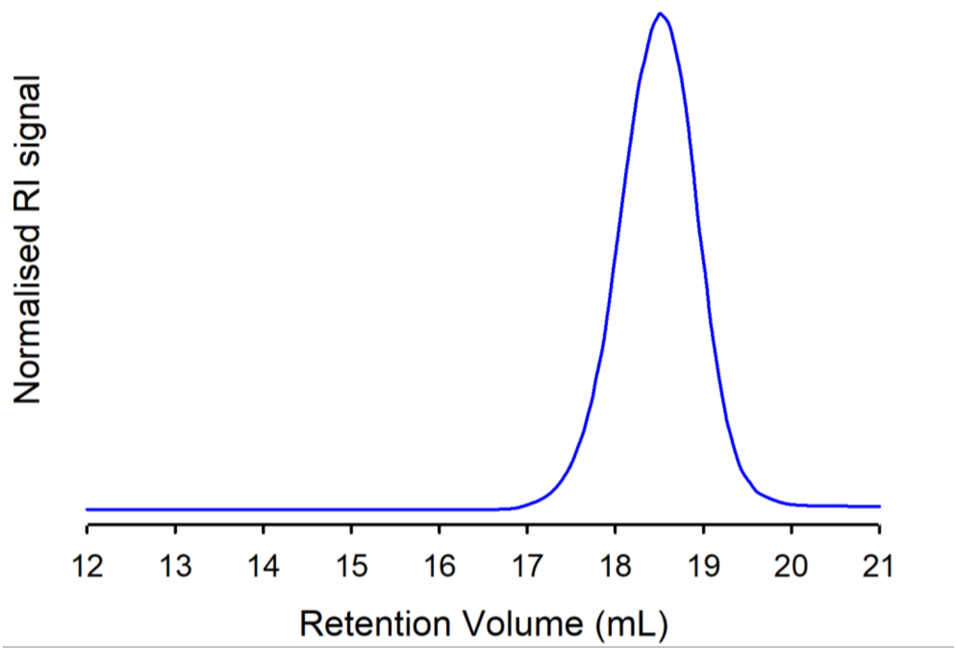

Figure S14. Triple detection size exclusion chromatography (RI chromatogram; THF/2% TEA eluent) of G_2-*p*(*t*BuMA$_{50}$) (homo LDH)

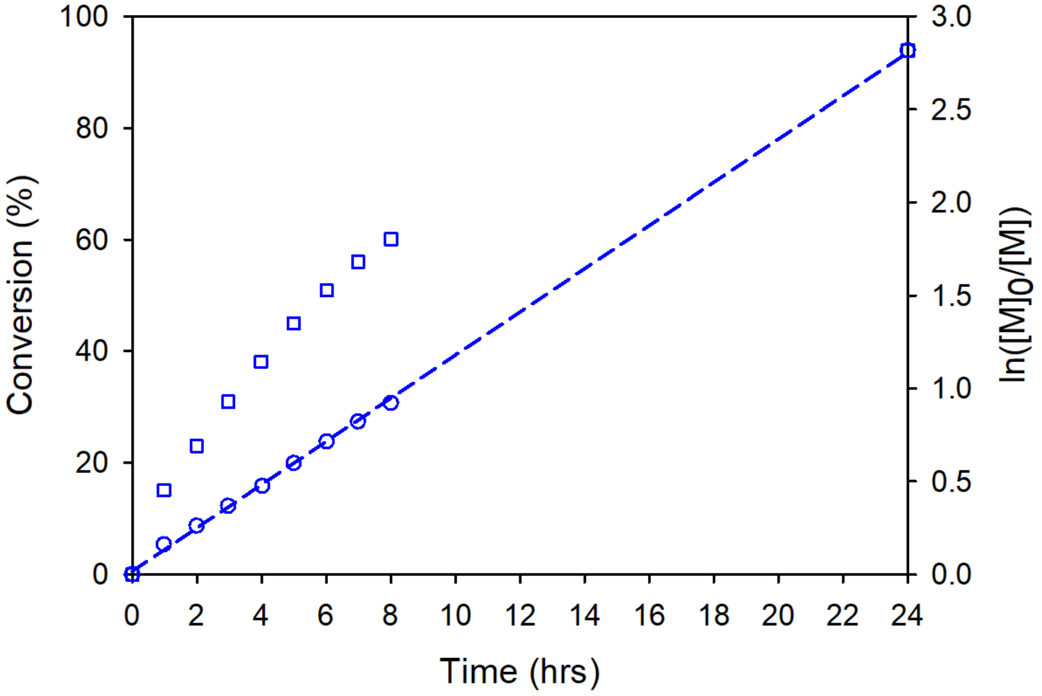

Figure S15. Kinetic analysis of the ATRP of G_2-*p*(*t*BuMA$_{50}$) (homo LDH). Open squares = conversion *vs.* time; open circles = semi-logarithm *vs.* time.

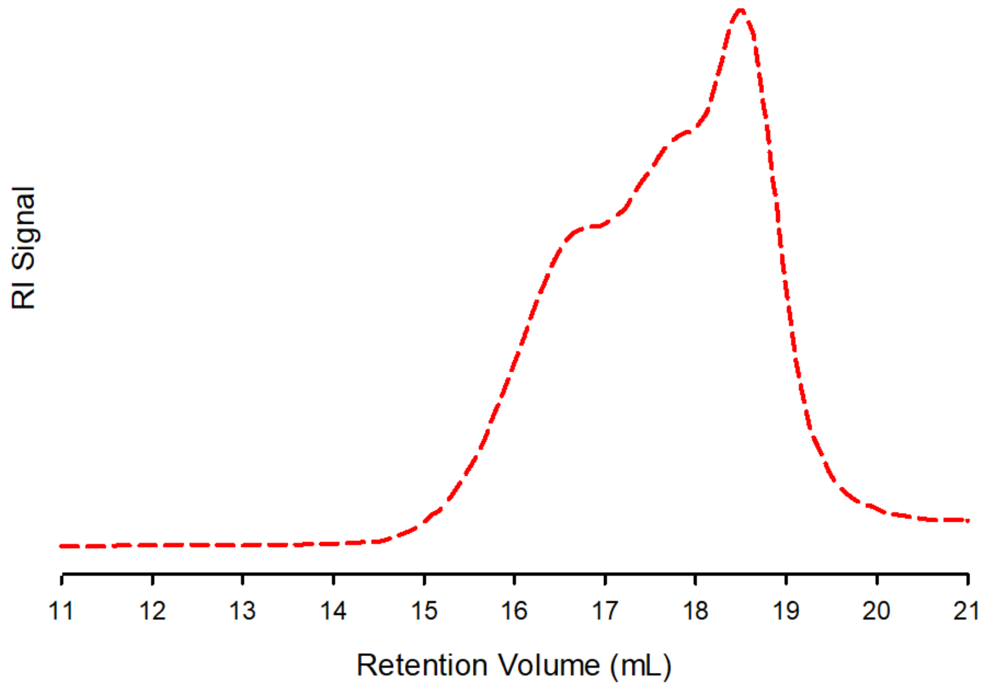

Figure S16. Triple detection size exclusion chromatography (RI chromatogram; THF/2% TEA eluent) of G2-*p*(*t*BuMA50-*co*-EGDMA0.95) (homo *hyp*-PD)

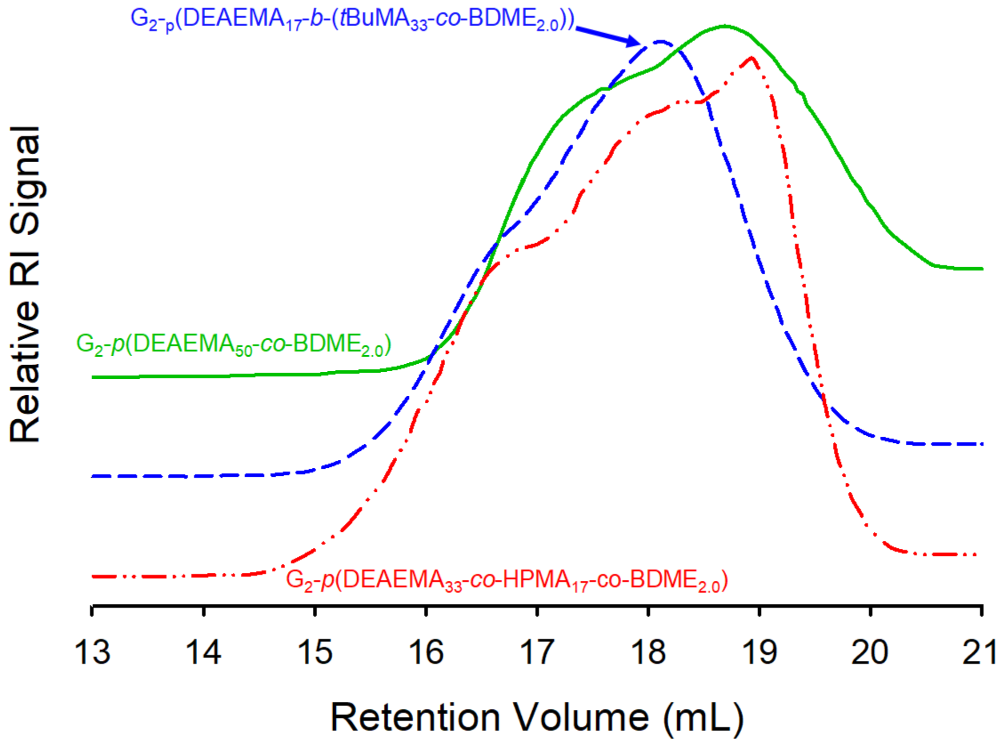

Figure S17. Triple detection size exclusion chromatography (overlaid RI chromatograms; THF/2% TEA eluent) of BDME containing *hyp*-PDs

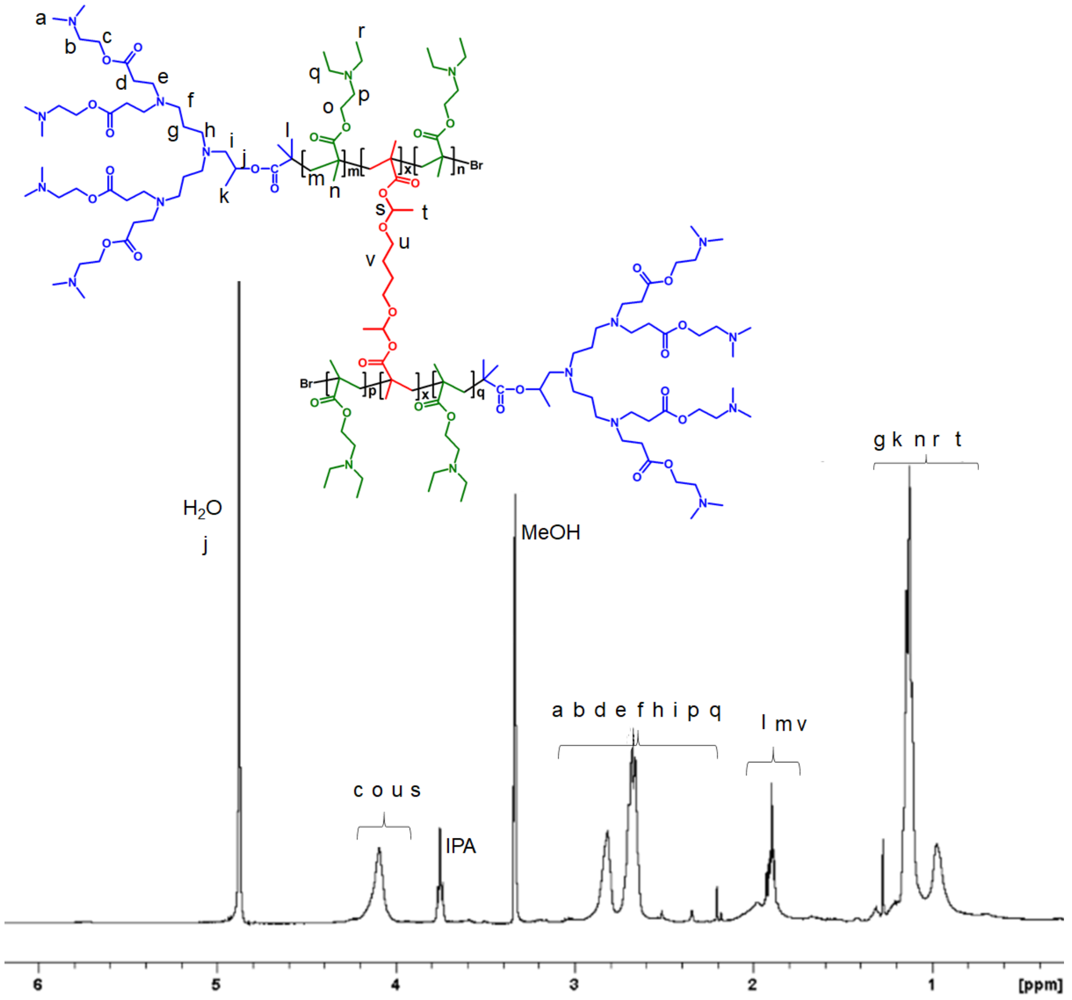

Figure S18. ^{1}H NMR spectrum (CD_3OD, 400 MHz) of G_2-*p*($DEAEMA_{50}$-*co*-$BDME_{2.0}$)) (homo *hyp*-PD)

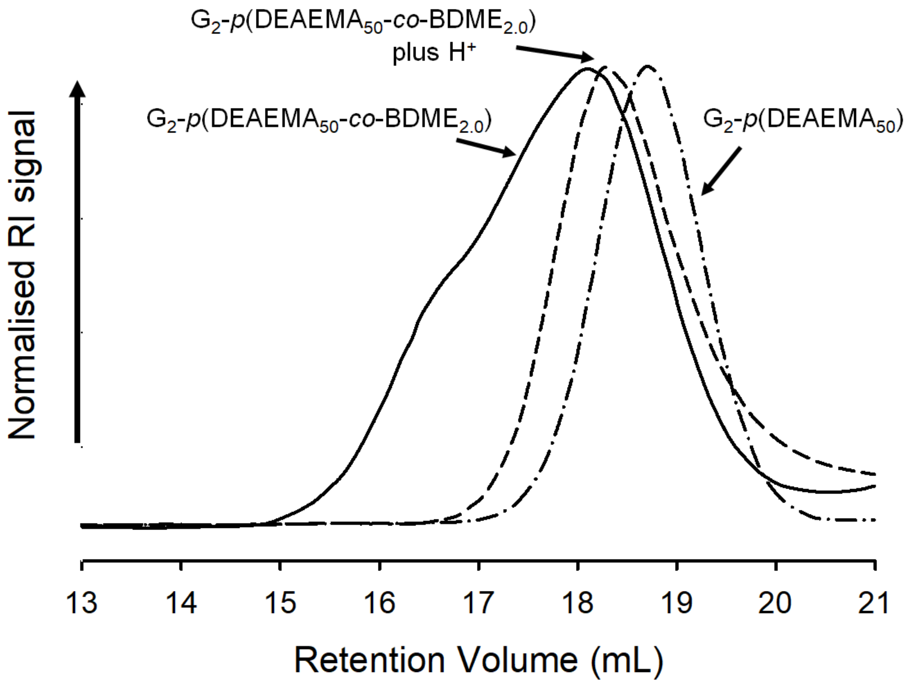

Figure S19. Triple detection size exclusion chromatography (overlaid RI chromatograms; THF/2% TEA eluent) of G_2-*p*($DEAEMA_{50}$-*co*-$BDME_{2.0}$) before addition of HCl to an acetone solution (solid line), after addition of HCl (dashed line) and corresponding G_2-*p*($DEAEMA_{50}$) homo LDH shown for comparison (dot-dash line)

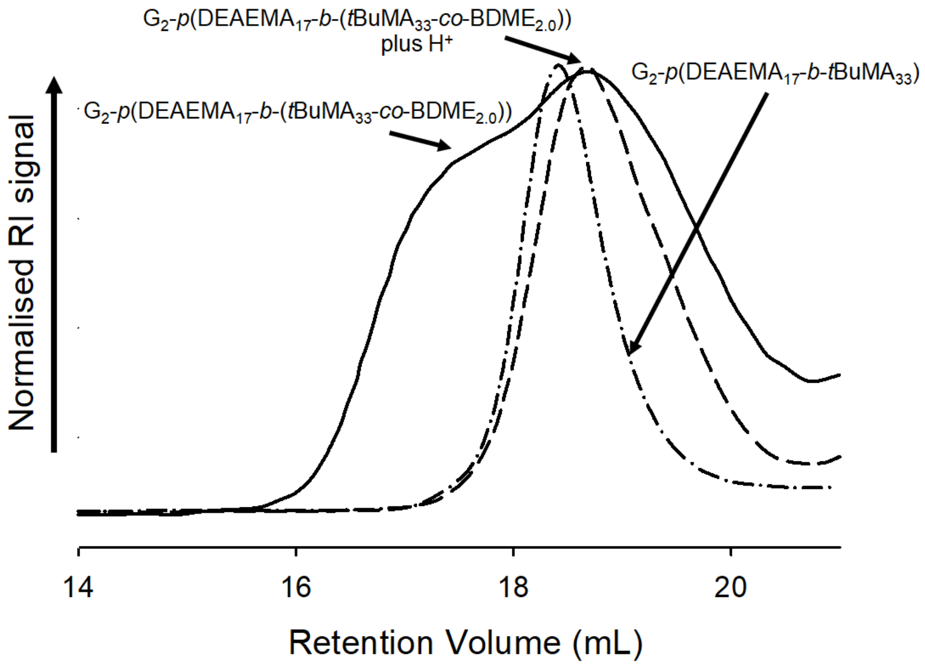

Figure S20. Triple detection size exclusion chromatography (overlaid RI chromatograms; THF/2% TEA eluent) of G_2-*p*($DEAEMA_{17}$-*b*-(*t*$BuMA_{33}$-*co*-$BDME_{2.0}$)) before addition of HCl to an acetone solution (solid line), after addition of HCl (dashed line) and corresponding G_2-*p*($DEAEMA_{17}$-*b*-*t*$BuMA_{33}$) block LDH shown for comparison (dot-dash line)

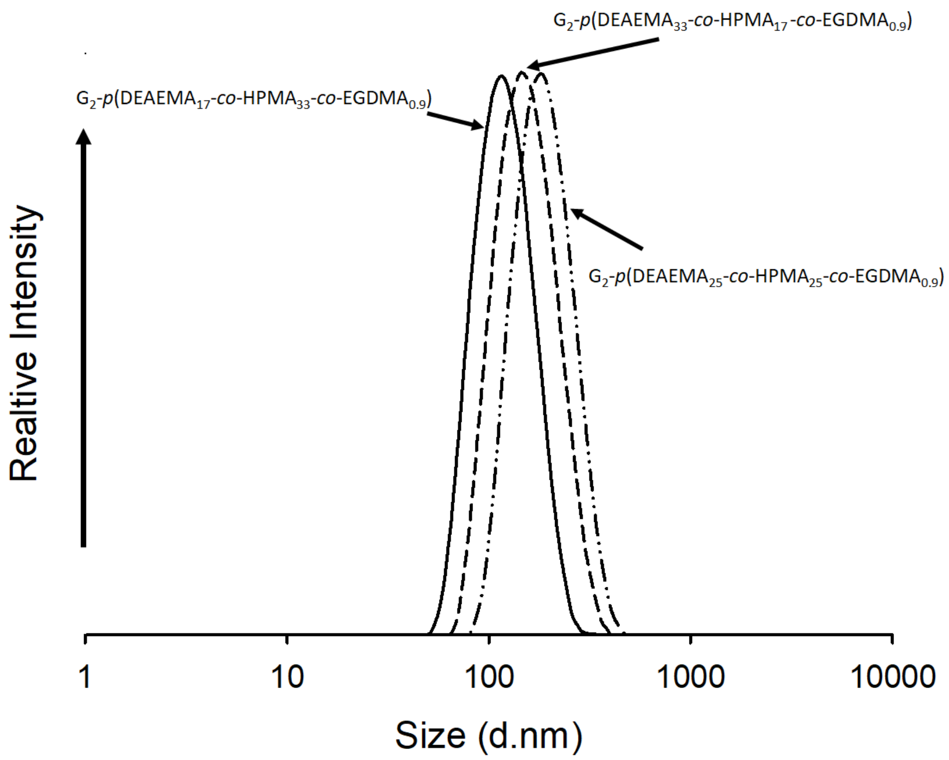

Figure S21. Dynamic light scattering analysis of nanoprecipitates (unfiltered) prepared from G_2-*p*($DEAEMA_y$-*co*-$HPMA_x$-*co*-$EGDMA_{0.9}$) statistical *hyp*-PDs

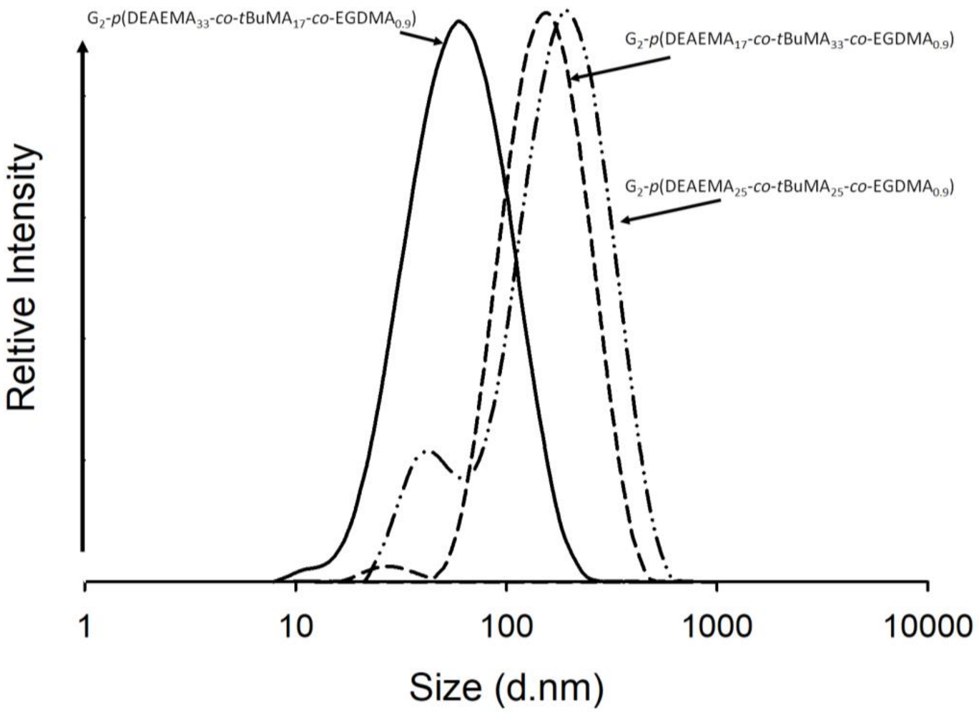

Figure S22. Dynamic light scattering analysis of nanoprecipitates (unfiltered) prepared from G_2-*p*($DEAEMA_y$-*co*-*t*$BuMA_x$-*co*-$EGDMA_{0.9}$) statistical *hyp*-PDs using an aqueous pH = 4 poor solvent environment

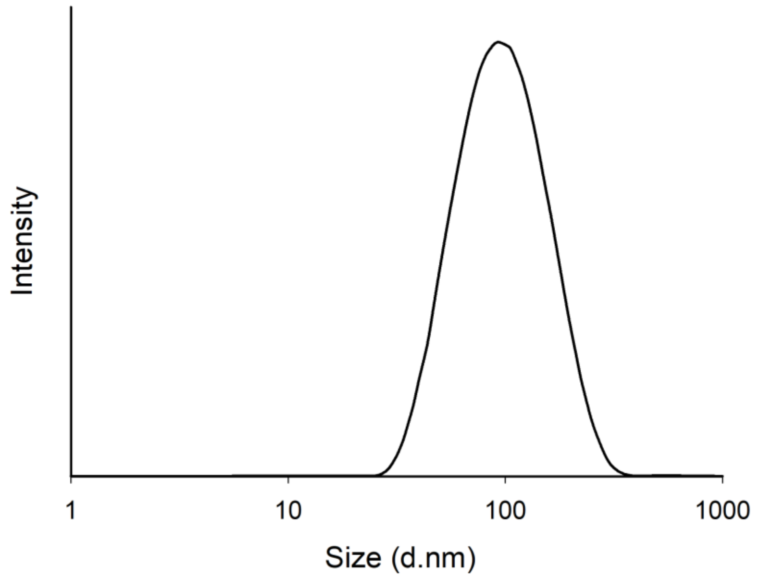

Figure S23. Dynamic light scattering analysis of nanoprecipitates (unfiltered) prepared from G_2-*p*($DEAEMA_{33}$-*b*-(*t*$BuMA_{17}$-*co*-$EGDMA_{0.9}$)) block *hyp*-PD using an aqueous pH = 4 poor solvent environment.

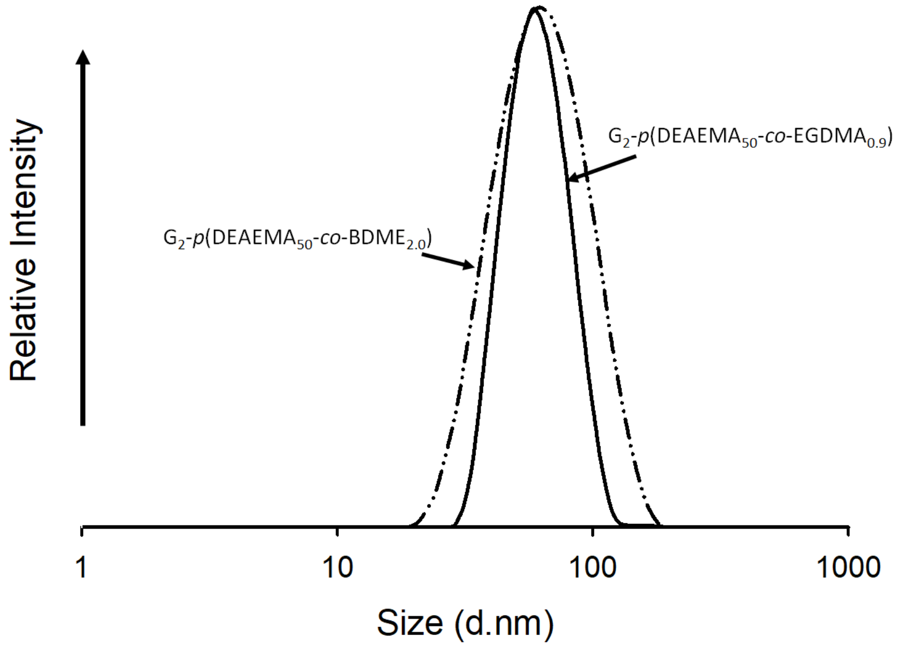

Figure S24. Dynamic light scattering analysis of nanoprecipitates (unfiltered) prepared from G_2-*p*($DEAEMA_{50}$-*co*-$EGDMA_{0.9}$) and G_2-*p*($DEAEMA_{33}$-*co*-$HPMA_{17}$-*co*-$EGDMA_{0.9}$) *hyp*-PDs

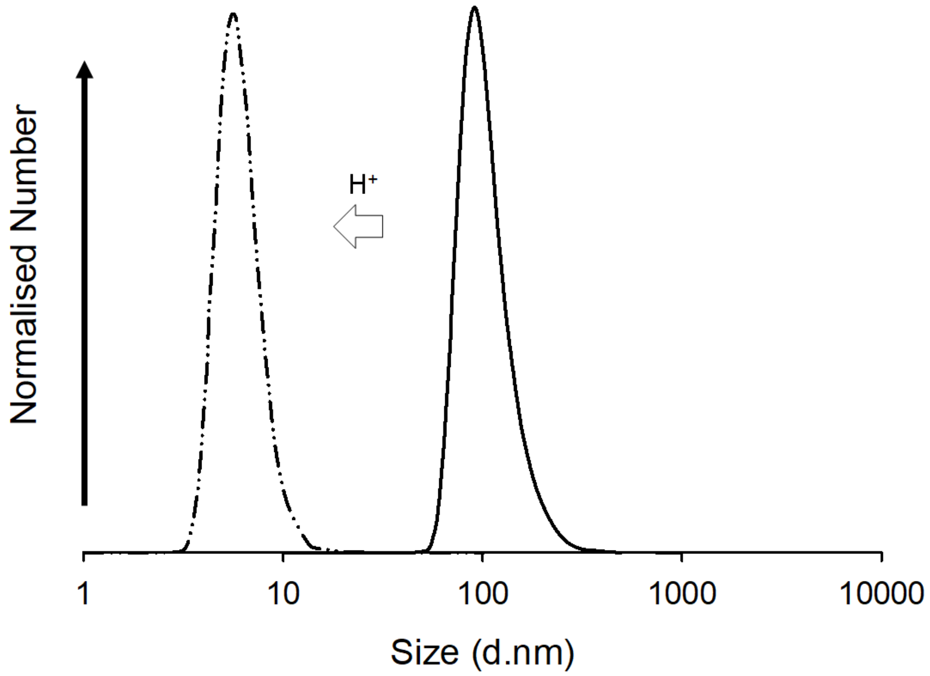

Figure S25. Dynamic light scattering analysis of nanoprecipitates (unfiltered; number distribution) showing the impact of acid addition on G_2-*p*($DEAEMA_{25}$-*co*-$HPMA_{25}$-*co*-$EGDMA_{0.9}$) statistical *hyp*-PD particles

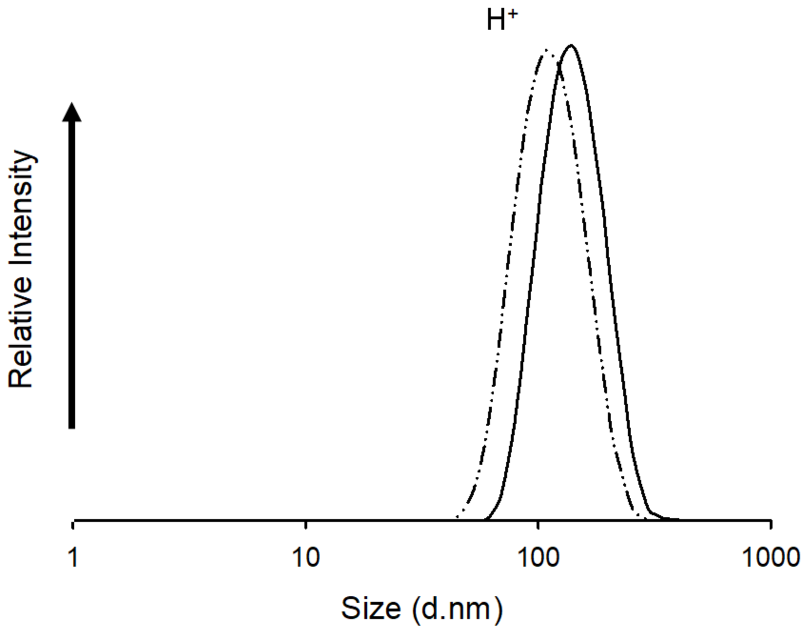

Figure S26. Dynamic light scattering analysis of nanoprecipitates (unfiltered) showing the impact of acid addition on G_2-*p*($DEAEMA_{17}$-*b*-(*t*$BuMA_{33}$-*co*-$BDME_{2.0}$) block *hyp*-PD particles

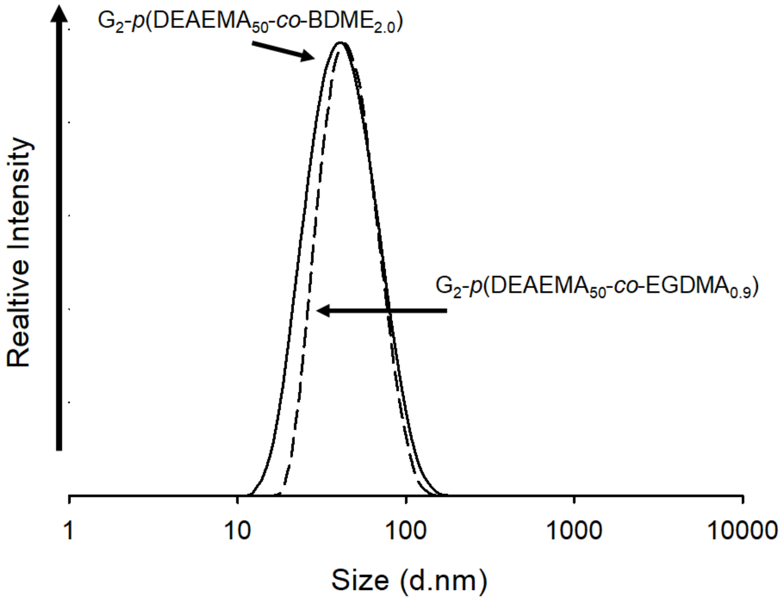

Figure S27. Dynamic light scattering analysis of nanoprecipitates (unfiltered) containing encapsulated fluoresceinamine, derived from G_2-*p*($DEAEMA_{50}$-*co*-$EGDMA_{0.9}$) or G_2-*p*($DEAEMA_{50}$-*co*-$BDME_{2.0}$).

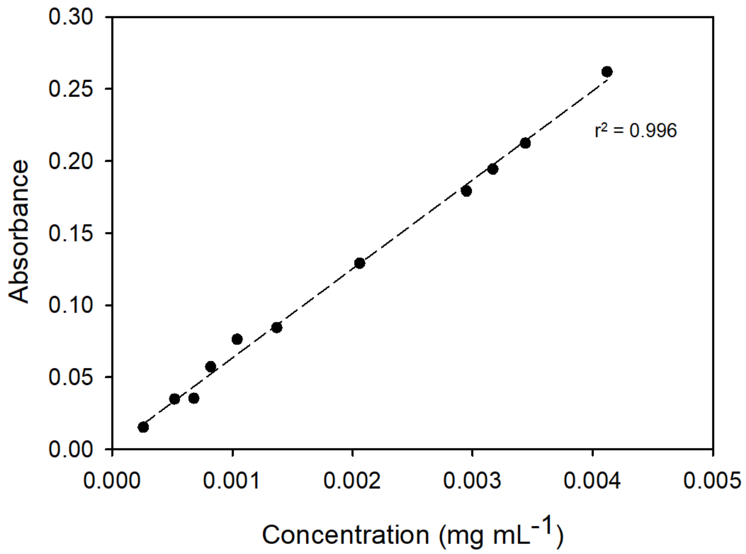

Figure S28. UV-visible spectroscopy calibration curve (acidic water) for determining fluoresceinamine concentration

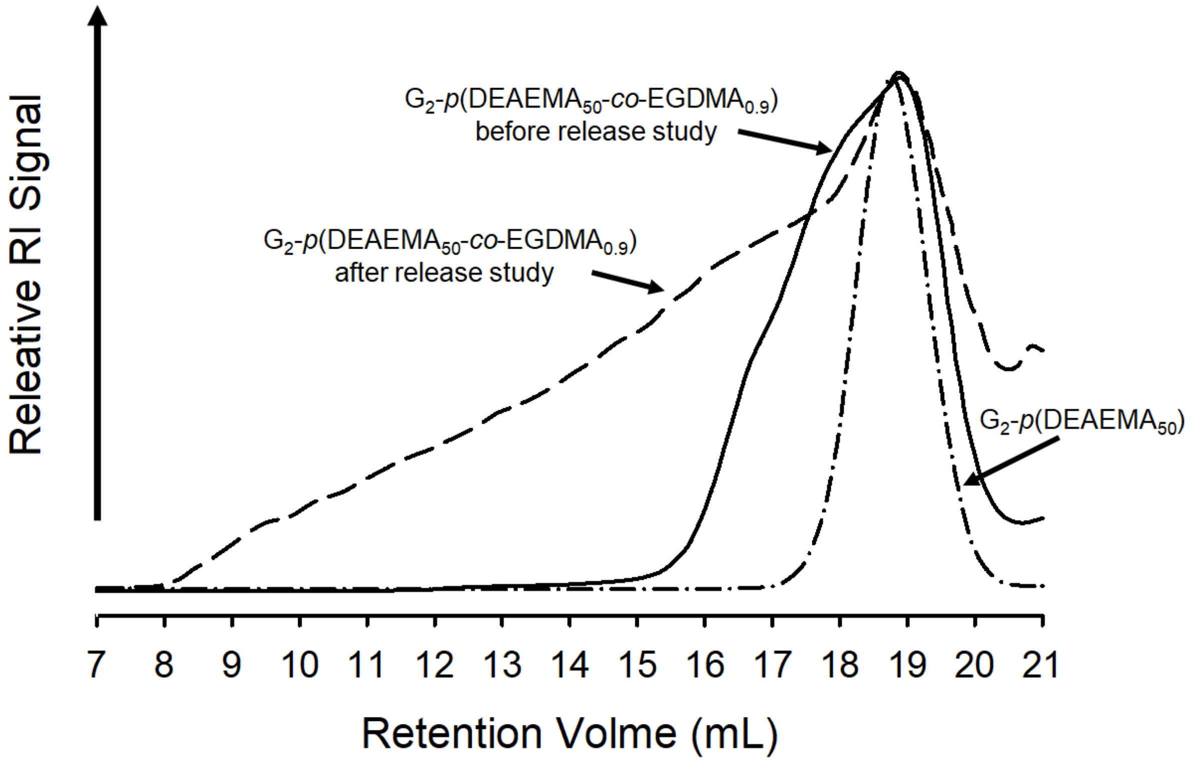

Figure S29. Triple detection size exclusion chromatography (overlaid RI chromatograms; THF/2% TEA eluent) of G_2-*p*(DEAEMA$_{50}$-*co*-EGDMA$_{0.9}$) before addition of HCl to an aqueous nanoprecipitate containing encapsulated fluoresceinamine (solid line), after addition of HCl and extraction/recovery of polymer (dashed line) and corresponding G_2-*p*(DEAEMA$_{50}$) homo LDH shown for comparison (dot-dash line).

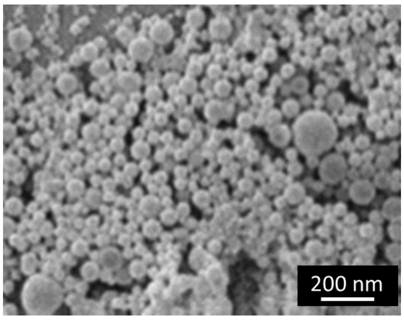

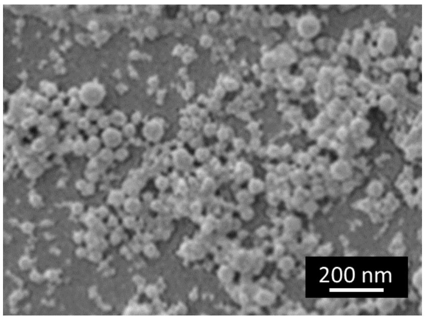

Figure S30. SEM images of G2-*p*(*t*BuMA$_{50}$-*co*-EGDMA$_{0.9}$) nanoprecipitates following nanoprecipitation into water (pH = 4) at 1 mg mL-1.

References

1. H. E. Rogers, P. Chambon, S. E. R. Auty, F. Y. Hern, A. Owen & S. P. Rannard, *Soft Matter*, 2015, 11, 7005-7015

2.E. Ruckenstein & H Zhang, *Macromolecules*, 1999, 32, 3979-3983
